# The relationship between HIV‐1 neuroinflammation, neurocognitive impairment and encephalitis pathology: A systematic review of studies investigating post‐mortem brain tissue

**DOI:** 10.1002/rmv.2519

**Published:** 2024-01-27

**Authors:** Monray Edward Williams, Petrus J. W. Naudé

**Affiliations:** ^1^ Human Metabolomics North‐West University Potchefstroom South Africa; ^2^ Department of Psychiatry and Mental Health University of Cape Town Cape Town South Africa; ^3^ Neuroscience Institute University of Cape Town Cape Town South Africa

**Keywords:** brain tissue, HIV‐associated neurocognitive disorders, markers, neuroinflammation, neuronal damage

## Abstract

The activities of HIV‐1 in the central nervous system (CNS) are responsible for a dysregulated neuroinflammatory response and the subsequent development of HIV‐associated neurocognitive disorders (HAND). The use of post‐mortem human brain tissue is pivotal for studying the neuroimmune mechanisms of CNS HIV infection. To date, numerous studies have investigated HIV‐1‐induced neuroinflammation in post‐mortem brain tissue. However, from the commonly investigated studies in this line of research, it is not clear which neuroinflammatory markers are consistently associated with HIV neurocognitive impairment (NCI) and neuropathology (i.e., HIV‐encephalitis, HIVE). Therefore, we conducted a systematic review of the association between neuroinflammation and NCI/HIVE from studies investigating post‐mortem brain tissue. Our aim was to synthesise the published data to date to provide commentary on the most noteworthy markers that are associated with NCI/HIVE. PubMed, Scopus, and Web of Science databases were searched using a search protocol designed specifically for this study. Sixty‐one studies were included that investigated the levels of inflammatory markers based on their gene and protein expression in association with NCI/HIVE. The findings revealed that the (1) transcript expressions of IL‐1β and TNF‐α were consistently associated with NCI/HIVE, whereas CCL2 and IL‐6 were commonly not associated with NCI/HIVE, (2) protein expressions of CD14, CD16, CD68, Iba‐1, IL‐1β and TNF‐α were consistently associated with NCI/HIVE, while CD45, GFAP, HLA‐DR, IL‐1 and IL‐6 were commonly not associated with NCI/HIVE, and (3) gene and protein expressions of CNS IL‐1β and TNF‐α were consistently associated with NCI/HIVE, while IL‐6 was consistently not associated with NCI/HIVE. These markers highlight the commonly investigated markers in this line of research and elucidates the neuroinflammatory mechanisms in the HIV‐1 brain that are involved in the pathophysiology of NCI/HIVE. These markers and related pathways should be investigated for the development of improved diagnostics, prognostics, and therapeutics of HAND.

**Abbreviations**
AAN: American Academy of Neurology
ACC: anterior cingulate cortex
AIF‐1: allograft inflammatory factor 1
AM: amygdala
ART: antiretroviral therapy
ARV: antiretroviral
BG: basal ganglia
CB: cerebellum
CCL: C‐C chemokine ligand
CCR: chemokine receptor
CD: cluster of differentiation
CNS: central nervous system
CSF: cerebrospinal fluid
CT: computed tomography
CXCL: chemokine (C‐X‐C motif) ligand
CXCR: C‐X‐C chemokine receptor type
DWM: deep white matter
eGFR: estimated glomerular filtration rate
ELISA: enzyme‐linked immunosorbent assay
FC: frontal cortex
FWM: frontal white matter
Gal: galectin
GFAP: glial fibrillary acidic protein
GP: globus pallidus
HAART: highly active antiretroviral therapy
HAD: HIV‐associated dementia
HAND: HIV‐associated neurocognitive disorders
HCV: hepatitis C
HIVE: HIV enecephalitis
HIVnE: HIV no encephalitis
HLA‐DR: human leucocyte antigen – DR isotype
HO: heme oxygenase
Iba‐1: ionised calcium‐binding adapter molecule
ICC: immunocytochemistry
IFN: interferon
IHC: immunohistochemistry
IL: interleukin
JBI: Joanna Briggs Institute
JNK: c‐Jun N‐terminal kinase
MAP: microtubule associated protein‐2
MAPK: mitogen‐activated protein kinase
MB: midbrain
MC: motor cortex
MCs: microglia cells
MED: medulla
MFC: midfrontal cortex
MFG: middle frontal gyrus
MGCs: multinucleated giant cells
MHC: major histocompatibility complex
MIG: monokine induced by gamma interferon
MIP: macrophage inflammatory protein
MMP: matrix metalloproteinases
MMSE: mini‐mental state examination
MN: microglia nodules
MND: mild neurocognitive disorder
MP: mononuclear phagocytes
MRI: magnetic resonance imaging
MSK: Memorial Sloan Kettering
N/A: not available
NCI: neurocognitive impairement or neurocognitively impaired
NCN: cognitive/cognitively normal
ND: neuronal damage
NE: neuropathological evaluation
NNTC: National NeuroAIDS Tissue Consortium
OC: occipital cortex
OPN: osteopontin
PAS: periodic acid‐Schiff
PCC: posterior cingulate cortex
PCR: polymerase chain reaction
PET: positron emission tomography
PI‐3: phosphatidylinositol‐3
PLWH: people living with HIV
PN: pons
PRIMSA: preferred reporting items for systematic reviews and meta‐analyses
RNA: ribonucleic acid
S100A8: S100 calcium‐binding protein MRP‐8
SAPK: stress‐activated protein kinase
SC: sensory cortex
SPC: spinal cord
TC: temporal cortex
TGF: transforming growth factor
TIMP: tissue inhibitors of metalloproteinases
TNFR: tumour necrosis factor receptor
TRAIL: tumour necrosis factor‐related apoptosis‐inducing ligand
UK: United Kingdom
VPR: Viral protein R
WM: white matter

## 1 INTRODUCTION

HIV‐1 is well known for its effects on the immune system, but it can also affect the central nervous system (CNS), leading to dysregulated inflammation and induction of neuronal damage and neurocognitive impairment (NCI) in people living with HIV (PLHW), defined as HIV‐associated neurocognitive disorders (HAND).^1^, ^2^ HIV‐1 is able to invade the CNS by what is known as the trojan horse hypothesis, whereby HIV‐1 is able to cross the blood‐brain barrier through infected mononuclear phagocytes that release viral particles into the brain parenchyma.^3^, ^4^, ^5^ Once within the CNS, the virus primarily infects microglia, the resident macrophages in the CNS.^6^ Studies with cell cultures and animals provide compelling evidence that HIV‐1 induces neuronal damage and cell death through direct and indirect mechanisms, leading to the development of HIV‐encephalitis (HIVE) neuropathology and clinical HAND.^7^, ^8^, ^9^

With the introduction of antiretroviral therapy (ART), the most severe form of HAND, HIV‐associated dementia (HAD), has dramatically declined.^2^, ^10^, ^11^ However, mild neurocognitive disorder and asymptomatic neurocognitive impairment have increased and are present in half of the population of PLWH.^2^ This persistent development of NCI in the modern ART era has been linked to a chronic inflammatory profile experienced by PLWH,^12^, ^13^ and neuroinflammation has been proposed as an important element in the pathophysiology of HAND.^14^, ^15^ Since access to brain tissue in living patients is unobtainable, studies are primarily limited to peripheral blood and cerebrospinal fluid (CSF) to explore the associations between neuroinflammatory markers with HAND in PLWH. In systematic reviews by our group, we have highlighted key immune markers associated with HAND in peripheral blood, that is, soluble cluster of differentiation (sCD14), sCD163, interleukin (IL)‐18 and C‐C motif chemokine ligand (CCL)2^16^ and CSF; sCD163, sCD14, Interferon (IFN)‐γ, IL‐1α, IL‐7, IL‐8, soluble tumour necrosis factor‐alpha receptor (sTNFR)‐II and IL‐6.^17^ These markers have consistently been associated with neurocognitive impairment in PLWH. Although these studies indicate the potential involvement of neuroinflammatory processes in neurocognitive impairments in PLWH, they do not accurately represent the neuroimmune regulation present in the CNS, particularly the regional expression of inflammatory markers and inflammatory phenotypes of immune cells in the brain (e.g., microglia and astrocytes). More recently, molecular imaging using positron emission tomography (PET) has enabled researchers to perform in vivo imaging of neuroinflammation in cognitively impaired PLWH by evaluating microglia activation with radiotracers specific for increased expression of the translocator protein 18 kDa (TSPO). A review of TSPO PET studies showed that literature on the presence of microglia activation in HIV remains inconclusive.^18^ The contradictory results from existing literature may be due to limitations including the limited number of study patients, nonspecific binding of the radiotracers, and the sensitivity of TSPO radiotracers to detect low‐grade neuroinflammation.

Post‐mortem brain tissue is the preferred material for investigating the involvement of CNS inflammation in HIV‐associated NCI and HIVE. HIV‐associated NCI is assessed and categorised through neuropsychological tests, while HIVE is evaluated and defined through post‐mortem brain tissue examination. Numerous studies have investigated inflammation in post‐mortem brain tissue of PLWH to date.^19^, ^20^ However, it is not clear which are the most commonly investigated markers in this line of research. Furthermore, there is currently no consensus on which of these commonly investigated markers are most consistently associated with NCI or HIVE. Identifying specific neuroinflammatory markers that are associated with NCI/HIVE, as revealed by studies with post‐mortem brain tissue, will enhance our understanding of the neuropathophysiology of HAND. Therefore, this study aims to summarise the current literature on HIV‐1‐induced neuroinflammation, neurocognitive performance, and neuropathology from studies investigating post‐mortem brain tissue.

## 2 METHODS

### 2.1 Study design

This is a descriptive and narrative systematic review aimed at summarising the extant literature on the association of neuroinflammatory markers and the presentation of neurocognitive impairment/pathology in studies investigating post‐mortem brain tissue. The study was conducted according to the Preferred Reporting Items for Systematic Reviews and Meta‐Analyses (PRISMA) guidelines. This study has been approved by the North‐West University Health Research Ethics Committee (NWU‐HREC): NWU‐00090‐23‐S1.

### 2.2 Eligibility criteria

The eligibility criteria for publications to be included in this study were those that investigated HIV‐positive treatment‐experienced adults (>18 years old, all medication types included and no cut‐off for treatment duration) with neuropsychological/neuropathological and medical assessments. The terms HIVE and HAND are often used interchangeably, however, this remains controversial.^9^ For this review, we considered participants to have (1) HIVE when diagnosed using histopathological analysis and (2) clinical NCI when diagnosed using a neuropsychological examination. It is important to note that the pathology of HIV‐associated encephalitis (HIVE) has various forms and classifications^9^, ^21^ and therefore, these factors were taken into consideration during the inclusion strategy. In order to ensure comparability, studies that included a histopathological diagnosis of HIVE were required to meet the criteria for ‘classic HIVE’,^17^ while other types of HIVE such as CD8+ T‐cell encephalitis and HIV leukoencephalopathy were excluded. In this review ‘classic HIVE’ was defined as presence of HIV‐infected cells in the brain, along with the formation of multi‐nucleated giant cells (MNG), microglial nodules (MN), microgliosis, astrogliosis, myelin pallor, and viral proteins such as p. 24.^22^ Hence, studies reporting HIVE were expected to report at least one of the criteria associated with ‘classic HIVE’.

For inclusion, all studies had to be investigating neuroinflammation from post‐mortem brain tissue. To ensure comparability across studies, inflammatory protein levels in post‐mortem brain tissue was required to be measured using enzyme‐linked immunosorbent assay (ELISA), immunohistochemistry (IHC), immunocytochemistry (ICC), or western blot. In addition, studies were also included if the gene cytokine expression level was measured using polymerase chain reaction (PCR). Considering that the presence of HIV‐1 in the brain can affect neuroinflammatory processes that are independent of neurocognitive impairment, our objective was to establish a link between dysregulated inflammation and neurocognitive impairment in HIV‐1, rather than solely the presence of the virus. Therefore, studies had to have a control group to be included, which may have been neurocognitively normal (NCN) or HIV non‐encephalitis (HIVnE) serving as negative controls for NCI and HIVE respectively. Exclusionary criteria were fundamental research studies with animals and cell culture models, and literature review studies. Studies investigating only serum and plasma markers were excluded, as these were considered outside the scope of this study. Findings from studies that investigated neuroinflammatory markers but did not report on the association between inflammation and NCI/HIVE were excluded.

### 2.3 Data sources

We electronically searched for publications in PubMed, Scopus and Web of Science databases based on all studies published until 24/05/2023. Eligible studies included published studies in English only. The search strategy was executed without publication date limitations. The full search criteria for each database are included in Supporting Information S1. The following search terms were applied to PubMed: (HIV [mh] OR HIV [tw] OR Acquired Immunodeficiency Syndrome [mh] OR ‘Acquired Immunodeficiency Syndrome’ [tw] OR AIDS [tw]) AND (HIV associated neurocognitive disorders [mh] OR HAND [tw] OR neurocognitive [tw] OR cogniti* [tw] OR Executive Function [mh] OR executive [tw] OR Memory [mh] OR memory [tw] OR Attention [mh] OR attention [tw] OR Neuropsychological Tests [mh] OR AIDS Dementia Complex [mh]) AND (Cytokines [mh] OR cytokin*[tw] OR Chemokines [mh] OR chemokine [tw] OR Inflammation [mh] OR inflammation [tw] OR Neurogenic Inflammation [mh] OR neuro‐inflammation [tw] OR TNF [tw] OR Interleukins [mh] OR interleukins [tw] OR Microglia [mh] OR microglia [tw] OR Monocytes [mh] OR monocyte* [tw] OR sCD163 [tw] OR sCD14 [tw] OR sCD40 [tw] OR CD68 [tw] OR Neopterin [mh] OR Interferons [mh] OR Ionised calcium binding adaptor molecule 1 [tw] OR IBA1 [tw] OR Glial Fibrillary Acidic Protein [tw] OR S100 Calcium Binding Protein beta Subunit [mh] OR CHI3L1 protein, human [mh]) AND (post‐mortem brain tissue [tw] OR postmortem brain OR Brain [mh] OR Immunohistochemistry [mh])

**FIGURE 1:**
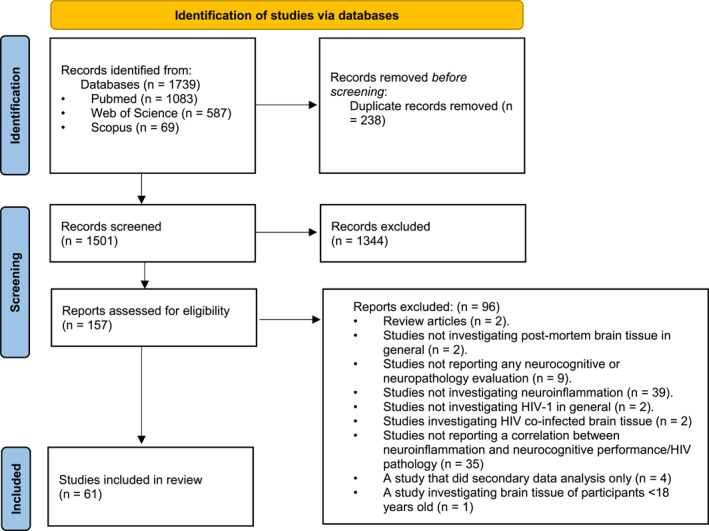
PRISMA 2020 flow diagram for new systematic reviews which included searches of databases and registers only.

In addition, we (1) reviewed reference sections of eligible articles and (2) manually searched for relevant publications. This search strategy and the retrieved articles are shown in Figure 1.

### 2.4 Data selection

All articles were retrieved and loaded onto a single database using a reference manager (EndNote X9, Clarivate). Two authors, MEW and PJWN independently identified studies meeting the inclusion criteria. Where there was a discrepancy in article inclusion/exclusion, this was discussed amongst all authors, and a decision was made regarding its suitability.

### 2.5 Quality assessment

The quality of the included studies was assessed by MEW. The quality criterion has been adopted from the Joanna Briggs Institute (JBI) critical appraisal tools. Here we have amended the JBI quality questions from the Checklist for Analytical Cross‐Sectional Studies^23^ by implementing a Likert‐type scale to provide a quantitative measure of study quality.^16^, ^24^, ^25^, ^26^, ^27^ For all included studies, we have collated the JBI quality questions which assess those factors, which may significantly affect the findings of the included studies (i.e., level of neuroinflammation and neurocognitive function). These included the questions:

(1) Confounders: Did the study report on potential confounders (e.g., substance misuse, comorbid conditions (e.g., Hepatitis C (HCV)), neurological conditions and psychiatric disorders), a relevant exclusionary criterion and were these controlled for upon statistical analysis?
(2) Study characteristics: Did the study report all key cohort information to contextualise the reported findings (i.e., age of participants at time of death, antemortem CD4^+^ count/viral load, use and duration of ART use)?
(3) NCI/HIVE diagnosis: Did the study report the antemortem NCI/HAND diagnosis criteria and/or did the study clearly define the criteria for HIVE post‐mortem?

Each question was rated for 0 = no, 1 = partly and 2 = yes. Studies that addressed all of the above questions and had a total rating of 6 were classified as high quality. Studies with a rating between 3 and 5 were considered intermediate‐quality and less than 3 as low quality (Supplementary Table S1).

### 2.6 Potential confounders

Plasma/CSF viral load,^28^ CD4^+^ count,^29^ and the HIV‐1 subtype^30^, ^31^ can affect immune marker levels and NCI. We, therefore, determined whether these confounders influenced the associations between the neuroinflammatory markers and NCI/HIVE that were reported by studies that were included in this review. First, we stratified studies according to viral load (Supplementary Table S2). We considered viral suppression as <2.6 log (50) copies/ml within blood/CSF as viral suppression^30^, ^31^, ^32^ and >2.6 log (50) copies/ml as non‐viral suppression. All studies reported viral load as a group mean (standard deviation) (Table 1). Second, we also stratified studies according to a mean/median CD4^+^ count of <200 cells/μL or >200 cells/μL (Supplementary Table S3). Mean values were primarily considered when stratifying studies. However, where the mean values were not available, the median values were used. Last, we wanted to determine if HIV subtype variation may have influenced the association between inflammatory markers and NCI/HIVE in post‐mortem brain tissue of PLWH, considering the fact that the HIV‐1 subtype variation and subtype‐specific viral protein amino acid substitutions can influence the prevalence of HAND,^91^, ^92^, ^93^, ^94^ as well as the levels of inflammatory markers.^95^, ^96^

**TABLE 1:** Cohort information of all included studies.

References	Cohort (*n*)	Age (years)	CD4 count (cells/μL)	Viral load (Plasma/CSF, log copies/ml)	Treatment status	Neuropsychological evaluation (NE) or neuropathological evaluation (HIVE)	Subtype/geographical location
33	People living with HIV (PLWH): 10 Neurocognitive impairment (NCI)/HIV encephalitis (HIVE): 8 Neurocognitive normal (NCN)/HIV non‐encephalitis (HIVnE): 2 HIV‐negative: 0 Female (%): N/A	PLWH: 40.1 (±6.4) NCI/HIVE: 39.4 (±6.3) NCN/HIVnE: 43 (±6)	N/A	N/A	N/A	N/A	N/A
34	PLWH: 20 NCI/HIVE: 11 NCN/HIVnE: 9 HIV‐negative: 10 Female (%): N/A	N/A	N/A	N/A	N/A	NE: a history of progressive cognitive and behavioural decline leading to marked impairment of occupational and social functions and usually associated with neurological motor deficit and a serial (at least three) neurological and neuropsychological examinations confirming progressive aggravation over weeks or months	N/A
35	PLWH: 36 NCI/HIVE: 8 NCN/HIVnE: 10 HIV‐negative: 5 Female (%): N/A	N/A	N/A	N/A	N/A	HIVE: Infiltration of macrophages, increase numbers of microglial cells, gliosis, myelin pallor and multinucleated giant cells (MGCs)	Edinburgh, United Kingdom (UK)
36	PLWH: 28 NCI/HIVE: 9 NCN/HIVnE: 19 HIV‐negative: 20 Female (%): 15	PLWH: 30 (±5) NCI/HIVE: 31.6 (±3.3) NCN/HIVnE: 30 (±5.6) HIV‐negative: 24.8 (±4.6)	PLWH: 139.4 (±149) NCI/HIVE: 30.65 (±31.32) NCN/HIVnE: 29.74 (±37.5) HIV‐negative: N/A	All groups >6	Highly active antiretroviral therapy (HAART) (0%)	N/A	Edinburgh, UK
37	PLWH: 45 NCI/HIVE: 27 NCN/HIVnE: 28 HIV‐negative: 9 Female (%): 37	PLWH: N/A NCI/HIVE: 33 (22–49) NCN/HIVnE: N/A HIV‐negative: 31 (18–45)	PLWH: N/A NCI/HIVE: 53 (1–137) NCN/HIVnE: N/A	All HAART‐treated cases <1.7	HAART (19%)	HIVE: p24 positivity in any region of the brain	Edinburgh, UK
38	PLWH: 27 NCI/HIVE: 15 NCN/HIVnE: 12 HIV‐negative: 10 Female (%): N/A	N/A	N/A	N/A	Zidovudine therapy, and combination antiretroviral therapy (cART) (14%)	N/A	Edinburgh, UK
39	PLWH: 15 NCI/HIVE: 7 NCN/HIVnE: 8 HIV‐negative: 5 Female (%): 16	PLWH: 41.2 (±9.3) NCI/HIVE: 45.3 (±9.2) NCN/HIVnE: 36.5 (±6.8) HIV‐negative: 63.4 (±15)	PLHW: All <300 cells/ul	N/A	ART (0%)	NE: Memorial Sloan Kettering (MSK) HIVE: p24 positive multinucleated giant cells were observed	N/A
40	PLWH: 17 NCI/HIVE: 15 NCN/HIVnE: 2 HIV‐negative: 0 Female (%): N/A	PLWH: 45.5 (±7.8) NCI: 44.9 (±8.1) NCN/HIVnE: 49.5 (±0.5)	N/A	N/A	N/A	NE: MSK	United States of America (USA)
41	PLWH: 74 NCI/HIVE: 56 NCN/HIVnE: 18 HIV‐negative: 0 Female (%): 16	PLWH: 47 (27–68) NCI: N/A NCN/HIVnE: N/A	PLWH: 53 (0–577) NCI: N/A NCN/HIVnE: N/A	PLWH: 4.25 (1.6–5.8) NCI: N/A NCN/HIVnE: N/A	N/A	NE: Frascati	California, USA
42	PLHW: 3 NCI/HIVE: 2 NCN/HIVnE: 1 HIV‐negative: 2 Female (%): N/A	N/A	N/A	N/A	N/A	NE: American Academy of Neurology (AAN)	N/A
43	PLHW: 15 NCI/HIVE: 8 NCN/HIVnE: 7 HIV‐negative: 4 Female (%): 16	PLHW: 41.3 (±7.6) NCI/HIVE: 39.87 (±7) NCN/HIVnE: 42.6 (±8) HIV‐negative: 49 (±7.9)	N/A	N/A	N/A	NE: Clinical diagnosis based on retrospective chart review at autopsy HIVE: Quantitation of microglial nodules (MN) and MGCs.	Manhattan, USA
8	PLWH: 15 NCI/HIVE: 9 NCN/HIVnE: 6 HIV‐negative: 8 Female (%): N/A	PLWH: 40.1 (±3.207) NCI/HIVE: 38.5 (±2.5) NCN/HIVnE: 42.5 (±2.7) HIV‐negative: 45.6 (±3.5)	N/A	N/A	HAART (20%)	HIVE: MSs and/or MGCs	Manhattan, USA
44	PLHW: 16 NCI/HIVE: 10 NCN/HIVnE: 6 HIV‐negative: 9 Female (%): 0	PLHW: 37.5 (±7.09) NCI/HIVE: 40 (±7) NCN/HIVnE: N/A HIV‐negative: 50 (±12)	N/A	N/A	N/A	N/A	USA
45	PLWH: 32 NCI/HIVE: 10 NCN/HIVnE: 22 HIV‐negative: 0 Female (%): 84	PLWH: 45.3 (±3.2 NCI/HIVE: 43 (±2.7) NCN/HIVnE: 46.3 (±2.83)	PLWH: N/A NCI/HIVE: 24 (18–147) NCN/HIVnE: N/A (4–150)	PLWH: N/A NCI/HIVE: 6.2 (4.2–6.4) NCN/HIVnE: N/A (1.8–5.8)	HAART (42%)	NE: AAN HIVE: The presence of HIV‐p24–positive cells, MNs, astrogliosis, and myelin pallor.	San Diego, USA
46	PLWH: 18 NCI/HIVE: 12 NCN/HIVnE: 6 HIV‐negative: 6 Female (%): N/A	PLWH: 45.2 (±9.3) NCI/HIVE: 43.4 (±8.6) NCN/HIVnE: 49 (±7.6) HIV‐negative: 50 (±7.6) Female (%): N/A	N/A	N/A	N/A	N/A	National NeuroAIDS tissue Consortium (NNTC), USA
47	PLWH: 11 NCI/HIVE: 7 NCN/HIVnE: 4 HIV‐negative: 8 Female (%): N/A	N/A	N/A	N/A	N/A	N/A	USA
48	PLHW: 13 NCI/HIVE: 8 NCN/HIVnE: 5 HIV‐negative: 0 Female (%): 23	PLHW: 44.9 (±4.79) NCI/HIVE: 43 (±5.5) NCN/HIVnE: 40 (±3)	PLHW: 111.5 (±79.2) NCI/HIVE: 60.5 (±37.1) NCN/HIVnE: 193 (±54)	PLHW: 4.9 (±0.79) NCI/HIVE: 4.8 (±0.64) NCN/HIVnE: 5.0560 (±1.0534)	N/A	N/A	San Diego, USA
49	PLHW: 83 NCI/HIVE: 25 NCN/HIVnE: 33 Other pathology: 25 HIV‐negative: 0 Female (%): 12	PLHW: 45.49 (±9.86) NCI/HIVE: N/A NCN/HIVnE: N/A	PLHW: 149.3 (±259.89) NCI/HIVE: NCN/HIVnE: 193	PLHW: 4.13 (±1.58) NCI/HIVE: NCN/HIVnE:	Antiretrovirals (ARVs) (36%)	NE: AAN HIVE: Presence of MNs, astrogliosis, HIV p24‐positive cells, and myelin pallor.	San Diego, USA
19	PLWH: 52 NCI/HIVE: 38 NCN/HIVnE: 14 HIV‐negative: 0 Female (%): 23	PLWH: 42.96 (±5.08) NCI: 45.05 (±2.89) NCN/HIVnE: 37.3 (±5.5)	PLWH: 98.6 (±175.02) NCI: 80.51 (±144.59) NCN/HIVnE: 147.7 (±239.01)	PLWH: 4.1 (±1.6) NCI: 4.02 (±1.58) NCN/HIVnE: 4.3 (±1.61)	ART (100%)	NE: AAN	NNTC, USA
50	PLWH: 13 NCI/HIVE: 9 NCN/HIVnE: 4 HIV‐negative: 6 Female (%): 20	PLWH: 42 (±5.6) NCI/HIVE: 43.7 (±4.9) NCN/HIVnE: 38 (±4.7) HIV‐negative: 48.4 (±15.8)	N/A	N/A	N/A	N/A	Manhattan, USA
51	PLWH: 12 NCI/HIVE: 9 NCN/HIVnE: 3 HIV‐negative: 5 Female (%): 20	PLWH: 41.8 (±5.8) NCI/HIVE: 43.8 (±4.9) NCN/HIVnE: 36 (±3.7) HIV‐negative: 47.2 (±14.9)	N/A	N/A	N/A	N/A	Manhattan, USA
52	PLWH: 41 NCI/HIVE: 24 NCN/HIVnE: 17 HIV‐negative: 0 Female (%): 15	PLWH: N/A NCI: 41 (N/A) NCN/HIVnE: 41 (N/A)	N/A	N/A	HAART (0%)	HIVE: Opaque periodic acid‐Schiff (PAS)‐positive HIV macrophages in the brain	N/A
53	PLWH: 51 NCI/HIVE: 34 NCN/HIVnE: 16 HIV‐negative: 0 Female (%): 9	PLWH: 38.6 (±8.6) HIVE/NCI: 37 (±6.89) NCN/HIVnE: 42 (±11)	N/A	N/A	N/A	NE: AAN and MSK	N/A
54	N/A	PLWH: 38 (±5) NCI: 4 (N/A) NCN/HIVnE: 5 (N/A) HIV‐negative: 42 (±4)	N/A	N/A	N/A	NE: MSK HIVE: Identification of macrophage infiltration and/or MGCs	USA
55	PLWH: 30 NCI/HIVE: 20 NCN/HIVnE: 10 HIV‐negative: 10 Female (%): N/A	N/A	N/A	N/A	N/A	N/A	USA
56	PLWH: 12 NCI/HIVE: 6 NCN/HIVnE: 6 HIV‐negative: 6 Female (%): 0	PLWH: 41.05 (±21.8) NCI/HIVE: 45.3 (±11.8) NCN/HIVnE: 36.8 (±8.6) HIV‐negative: 32 (±9)	PLWH: 13.35 (±22.91) NCI/HIVE: 22.5 (±29.4) NCN/HIVnE: 4.2 (±4.6) HIV‐negative: N/A	N/A	N/A	NE: Mini‐mental state examination (MMSE)	N/A
57	PLWH: 44 NCI/HIVE + neuronal damage (ND): 14 NCI/HIVE + no ND:10 No NCI/HIVE + ND:10 No NCI/HIVE + no ND: 10 Female (%): N/A	PLWH: (28–54) (N/A) NCI/HIVE + neuronal damage (ND): 41.0 (±1.6) NCI/HIVE + no ND: 41.5 (±2.3) No NCI/HIVE + ND: 39.0 (±3) No NCI/HIVE + no ND: 41.0 (±2.5)	N/A	N/A	N/A	HIVE: The presence of MGCs, HIV‐infected microglial cells and viral load	San Diego, USA
58	PLHW: 80 NCI/HIVE: 60 NCN/HIVnE: 20 HIV‐negative: 0 Female (%): 18	PLHW: 46.6 (±9) NCI/HIVE: N/A NCN/HIVnE: N/A	PLHW: 85 (±110.7) NCI/HIVE: N/A NCN/HIVnE: N/A	PLHW: 3.9 (±1.4) NCI/HIVE: N/A NCN/HIVnE: N/A	cART (N/A)	NE: Frascati	California, USA
59	PLHW: 175 NCI/HIVE: 135 NCN/HIVnE: 50 HIV‐negative: 0 Female (%): 18.6	PLHW: 47.4 (±9.2) NCI/HIVE: N/A NCN/HIVnE: N/A	PLWH: 122 (±168) NCI/HIVE: N/A NCN/HIVnE: N/A	PLWH: 4.2 (N/A) NCI/HIVE: N/A NCN/HIVnE: N/A	cART (N/A)	NE: Frascati	USA
60	PLWH: 23 NCI/HIVE: 23 HIV‐negative: 7 Female (%): N/A	All participants: 21–40 (N/A) 21–40 years	N/A	N/A	N/A	HIVE: The presence of MGCs	San Diego, USA
61	PLWH: 17 NCI/HIVE: 9 NC: 8 Female (%): N/A	PLWH: 41.41 (±3.06) NCI/HIVE: 42.5 (±2.3) NCN/HIVnE: 40.2 (±3.5)	N/A	PLWH: 6.45 (±6.45) NCI/HIVE: 5.75 (±4.8) NC: 2.4 (±2.1)	N/A	HIVE: The presence of HIV in the brain, activated microglia, MGCs, astrogliosis and myelin pallor.	San Diego, USA
62	PLWH: 20 NCI/HIVE: 8 NCN/HIVnE: 12 HIV‐negative: 0 Female (%): 10	PLWH: 38.2 (±3.35) NCI: 39.4 (±4.3) NCN/HIVnE: 37.6 (±2.5)	PLWH: 71.96 (±44.69) NCI: 47.6 (±27.3) NCN/HIVnE: 88.2 (±47.5)	N/A	N/A	NE: AAN	N/A
63	PLWH: 7 NCI/HIVE: 4 NCN/HIVnE: 3 HIV‐negative: 5 Female (%): N/A	PLWH: 25.84 (±12.3) NCI: 24.75 (±20.4) NCN/HIVnE: 27.3 (±15.79) HIV‐negative: N/A	NCI: All <34 NCN/HIVnE: All <30	N/A	N/A	N/A	N/A
64	PLWH: 10 NCI/HIVE: 6 NCN/HIVnE: 4 HIV‐negative: 5 Female (%): 20%	PLWH: 39.8 (±10.2) NCI: 63.4 (±15) NCN/HIVnE: 32.3 (±3.8) HIV‐negative: 44 (±10)	PLWH: All <300	N/A	Treatment naïve (100%)	NE: MSK	N/A
65	PLWH: 6 NCI/HIVE: 4 NCN/HIVnE: 2 HIV‐negative: 3 Female (%):	PLWH: 38.16 (±15.15) NCI/HIVE: 30.75 (±10.91) NCN/HIVnE: 53 (±11) HIV‐negative: 61 (±8.16)	All groups <200	N/A	N/A	HIVE: Presence of MGCs.	N/A
66	PLWH; 14 NCI/HIVE: 9 NCN/HIVnE: 5 HIV‐negative: 5 Female (%): N/A	N/A	All <200	N/A	N/A	HIVE: Human Leucocyte antigen – DR isotype (HLA‐DR) microglia, HIV‐1 infection of ramified microglia, formation of MN and infiltrating macrophages and abundant microglia cells (MCs)	N/A
67	PLWH: 22 NCI/HIVE: 11 NCN/HIVnE: 11 HIV‐negative: 10 Female (%): 40	PLWH: 37.2 (±13.9) NCI/HIVE: 35 (±9) NCN/HIVnE: 39 (±18) HIV‐negative: 48 (±19)	N/A	N/A	ART (22%)	HIVE: Presence of the characteristic MGCs and HIV gp41.	N/A
68	PLWH; 21 NCI/HIVE: 10 NCN/HIVnE: 11 HIV‐negative: 7 Female (%): N/A	PLWH: 37.23 (±13.9) NCI/HIVE: 33.7 (±7.6) NCN/HIVnE: 40.16 (±17) HIV‐negative: N/A	N/A	N/A	ART (40%)	HIVE: Presence of the characteristic MGCs and HIV gp41.	N/A
69	PLHW: 30 NCI/HIVE: 15 NCN/HIVnE: 13 HIV‐negative: 16 Female (%): N/A	PLHW: 40 (±11.6) NCI/HIVE: 40 (±12) NCN/HIVnE: 40 (±12) HIV‐negative: 42.9 (±14.4)	PLHW: 271 (±129.7) NCI/HIVE: 37 (±52) NCN/HIVnE: 108 (±160) HIV‐negative: N/A	N/A	N/A	HIVE: HIV‐1 seropositivity, (2) history of progressive cognitive and behavioural decline, neurological and/or neuropsychological findings consistent with a decline from the premorbid baseline, and (4) opportunistic processes in the NCN/HIVnES excluded by CT or MRI and cerebrospinal fluid (CSF) analysis.	USA
70 ^a^	PLWH: 18/12 NCI/HIVE: 5/5 NCN/HIVnE: 13/7 HIV‐negative: 6/5 Female (%): N/A/12%	PLWH: N/A NCI/HIVE: 43 (37–43) NCN/HIVnE: 52 (43–53.5) HIV‐negative: 48 (48‐ 48)	PLWH: N/A NCI/HIVE: 40 (21–78) NCN/HIVnE: 85 (75–295)	PLWH: N/A NCI/HIVE: 5.30 (4.7–5.4) NCN/HIVnE: 2.25 (1.7–2.51)	N/A	N/A	USA
71	PLHW: 7 NCI/HIVE: 4 NCN/HIVnE: 3 HIV‐negative: 4 Female (%): 9	PLHW: 42.3 (±5.8) NCI/HIVE:38.8 (±4.7) NCN/HIVnE: 47 (±4.3) HIV‐negative: 46.5 (±8.4)	N/A	PLWH: 6.55 (±6.6)NCI/HIVE: 6.75 (±6.6)NCN/HIVnE: 4.95 (±5.05)	N/A	HIVE: HLA‐DR microglia, HIV‐1 infection of ramified microglia, formation of MN and infiltrating macrophages and abundant MCs.	Washington DC, USA
72	PLWH: 17 NCI/HIVE: 13 NCN/HIVnE: 4 HIV‐negative: 2 Female (%): NA	N/A	N/A	N/A	N/A	NE: AAN and MSK HIVE: Presences of MGCs and gp41	UK
73	PLWH: 17 NCI/HIVE: 9 NCN/HIVnE: 8 HIV‐negative: 5 Female (%): NA	N/A	N/A	N/A	N/A	NE: AAN and MSK HIVE: Presence of MGCs	UK
74	PLWH: 8 NCI/HIVE: 5 NCN/HIVnE: 3 HIV‐negative: 5 Female (%): 7	PLWH: 40.4 (±5) NCI/HIVE: 39.6 (±2.3) NCN/HIVnE: 41.6 (±7.6) HIV‐negative: 41.6 (±9.1)	N/A	N/A	N/A	N/A	USA
75	PLWH: 74 NCI/HIVE: 44 NCN/HIVnE: 30 HIV‐negative: 35 Female (%): 7	PLWH: 15–66 years NCI/HIVE: N/A NCN/HIVnE: N/A HIV‐negative: N/A	N/A	N/A	N/A	N/A	Austria
76	PLWH: 12 NCI/HIVE: 3 NCN/HIVnE: 4 HIV‐negative: 6 Female (%): 0	PLHW: 41 (±3) years	PLWH: 30 (±11)	N/A	N/A	NE: MMSE and test battery HIVE: Histology combined with gp41 immunoreactivity.	France
77	PLWH: 12 NCI/HIVE: 3 NCN/HIVnE: 4 HIV‐negative: 6 Female (%): 0	PLHW: 41 (±3) years	PLWH: 30 (±11)	N/A	N/A	NE: MMSE HIVE: Astrogliosis; gp41‐positive cells; the severity of cognitive decline measured by the MMSE	France
78	PLHW: 14 NCI/HIVE: 14 NCI other: 5 NCN/HIVnE: N/A HIV‐negative: 15 Female (%): 34	PLWH: 41.57 (±8.9) HIV‐negative: 48.6 (±6.9)	PLWH: 91.78 (±98.38)	PLWH: 5.2 (±5.45)	Antiretroviral (ARV, 100%)	NE: AAN	USA
79	PLWH: 13 NCI/HIVE: 6 NCI other: 3 NCN/HIVnE: 4 HIV‐negative: 4 Female (%): N/A	PLWH: 24–48 (N/A) NCI/HIVE: N/A NCI other: N/A NCN/HIVnE: N/A HIV‐negative: 33–50 (N/A)	N/A	N/A	N/A	HIVE: Presence of MGCs and MNs	Los‐Angeles, USA
80	PLWH: 28 NCI/HIVE: 16 NCN/HIVnE: 12 HIV‐negative: 5 Female (%): N/A	N/A	N/A	N/A	N/A	N/A	USA
20	PLHW: 47 NCI/HIVE: 39 NCN/HIVnE: 8 HIV‐negative: 0 Female (%): 13	PLHW: 45.5 (±9.43) NCI: 47.2 (±9.19) NCN/HIVnE: 37.3 (±5.7)	PLHW: 154.4 (±173.52) NCI: 155.79 (±160.97) NCN/HIVnE: 147.7 (±239.01)	PLHW: 5.1 (±1.58) NCI: 3.8 (±1.58) NCN/HIVnE: 4.3 (±1.61)	ART	NE: AAN	San Diego, USA
81	PLHW: 18 NCI/HIVE: 8 NCN/HIVnE: 10 HIV‐negative: 5 Female (%): 50	PLHW: 44.6 (±8.59) NCI: 45.5 (±3.5) NCN/HIVnE: 44.75 (±12.1) HIV‐negative: 45.8 (±16.9)	PLHW: 52.17 (±93.1) NCI: 17.25 (±31.07) NCN/HIVnE: 80.1 (±114.39)	PLHW: 5.8 (±6.1) NCI: 6 (±6.2) NCN/HIVnE: 5.3 (±5.4)	cART (44%)	NE: AAN	Manhattan, USA
82	PLWH: 8 NCI/HIVE: 4 NCN/HIVnE: 4 HIV‐negative: 4 Female (%): 42	PLWH: 33 (±16.69) NCI/HIVE: 33 (±17) NCN/HIVnE: 33 (±19) HIV‐negative: 45 (±13)	N/A	N/A	N/A	N/A	N/A
83	PLWH: 10 NCI/HIVE: 5 NCN/HIVnE: 5 HIV‐negative: 0 Female (%): N/A	PLWH: N/A NCI/HIVE: 35.8 (±3.9) NCN/HIVnE: N/A	PLWH: N/A NCI/HIVE: 25.4 (±16.4) NCN/HIVnE: N/A	PLWH: N/A NCI/HIVE: 5.65 (±5.35) NCN/HIVnE: N/A	AZT treatment (100%)	HIVE: Perivascular infiltration by mononuclear cells	Buenos Aires, Argentina
84	PLWH: 24 NCI/HIVE: 10 NCN/HIVnE: 14 HIV‐negative: 9 Female (%): N/A	PLWH: 39.6 (±13.4) NCI: 39.8 (±13) NCN/HIVnE: 39.9 (±15) HIV‐negative: 42 (±11)	PLWH: 56.75 (±37.2) NCI: 55 (±57) NCN/HIVnE: 58 (±14)	N/A	N/A	NE: MSK	N/A
85	N/A	N/A	N/A	N/A	N/A	HIVE: HIV‐1 gp41 antigen immunoreactivity in brain sections that showed typical neuropathological alterations	USA
86	PLWH: 20 NCI/HIVE: 11 NCN/HIVnE: 9 HIV‐negative: 2 Female (%): N/A	N/A	N/A	N/A	Treatment naïve (100%)	HIVE: Perivascular infiltration, presence of p24, MGCs, or present along with MNs	Vienna
87	PLHW: 12 NCI/HIVE: 6 NCN/HIVnE: 6 HIV‐negative: 6 Female (%): N/A	N/A	N/A	N/A	N/A	N/A	N/A
88	PLWH: 24 NCI/HIVE: 17 NCN/HIVnE: 7 HIV‐negative: 15 Female (%): 31	PLWH: 45 (±10) NCI: 45,3 (±8.5) NCN/HIVnE: 47.4 (±9.9) HIV‐negative: 52 (±10)	PLWH: 112 (±175.2) NCI: 112.1 (±175.2) NCN/HIVnE: 93.7 (±151.8)	PLWH: 5.4 (±5.5)/4.8 (±5.3) NCI: 5.4 (±5.48)/5 (±5.39) NCN/HIVnE: 5.46 (±5.48)/3.98 (±4.21)	N/A	NE: Test battery measuring 7 domains	Manhattan, USA
89	PLWH: 5 NCI/HIVE: 5 HIV‐negative: 3 Female (%): 29	PLWH: 41.4 (±6.7) NCI/HIVE: 41.4 (±6.7) HIV‐negative: 34 (±9.2)	N/A	N/A	N/A	NE: Retrospective chart review at autopsyHIVE: Quantitation of MN and MGCs	Manhattan, USA
90	PLWH: 6 NCI/HIVE: 2 NCN/HIVnE: 4 HIV‐negative: 0 Female (%): 16	PLWH: 40.7 (±18.4) NCI/HIVE: 23.5 (±20.5) NCN/HIVnE: 40.7 (±18.4)	N/A	PLWH: 5.55 (±5.4) NCI/HIVE: 5.53 (±4.5) NCN/HIVnE: 5.58 (±5.56)	AZT, DDI (50%)	NE: The American Academy of Neurology	Sydney, Australia

Abbreviations: AAN, American Academy of Neurology; ARV, Antiretroviral; CSF, cerebrospinal fluid; CT, computed tomography; HAART, Highly active antiretroviral therapy; HIVE, HIV enecephalitis; HIVnE: HIVE no encephalitisHLA‐DR, Human Leucocyte; Antigen – DR isotype, MCs, microglia cells; MGCs, Multinucleated giant cells; MMSE, Mini Mental State Examination; MN, Microglia nodules; MRI, magnetic resonance imaging; MSK, Memorial Sloan Kettering; N/A, Not available; NCI, Neuroongitve impairement or Neurocognitively impaired; NCN: Neurocogntive(ly) normalND: Neuronal damage, NE, Neuropathological evaluation; NNTC, National NeuroAIDS Tissue Consortium; PAS, periodic acid‐Schiff; PLWH, people living with HIV; UK, United Kingdom; USA, United States of America.

^a^ Study presents secondary data analysis and primary data analysis and reported as Secondary/primary data within Table 1 (Premeaux et al., 2019).

## 3 RESULTS

### 3.1 Study characteristics

Using this criterion and search strategy, 1739 abstracts and titles were screened as indicated in Figure 1. Duplicates (*n* = 238) were removed, resulting in 1501 studies. Thereafter, abstracts and titles were screened and a total of 1344 studies were removed. A 157 full‐text articles were assessed for eligibility, and 96 articles we excluded. The reasons for exclusion are provided in Figure 1. Using this criterion, a total of 61 studies were included for data extraction. Cohort information was available for *n* = 59/61 (97%) of studies and across all studies a total sample size of *n* = 1546 PLWH, *n* = 949 NCI/HIVE, *n* = 562 NCN and *n* = 321 HIV‐negative controls were included. The majority of studies (*n* = 48, 79%) have reported the age of the study participants, with the mean/median ages ranging from 21 to 56 years old. The majority of the participants were male (Table 1).

### 3.2 Neuropsychological/neuropathological evaluation

The studies that were included reported the type of antemortem neuropsychological evaluation that was used to classify NCI (*n* = 17) and/or the post‐mortem neuropathological evaluation used to diagnose HIVE (*n* = 18). Certain studies included neuropsychological and neuropathological evaluation (*n* = 9). All studies that included HIVE pathological diagnosis were according the ‘classic HIVE’ criteria.^22^ Seventeen studies have not reported the type of neuropsychological evaluation or neuropathological evaluation used to diagnose NCI or HIVE in PLWH (Table 1).

### 3.3 Quality of assessment of studies

Most of the included studies were rated as intermediate quality (*n* = 39), with four considered as high quality and 18 as low quality. Only *n* = 4 studies received a total score of 6 for the overall quality criteria (Supplementary Table S1). The majority of studies did not report all relevant study confounders (*n* = 49) and key study characteristics (e.g., CD4^+^ counts and viral loads, *n* = 54). The majority of studies have reported some type of neuropsychological/neuropathological evaluation for NCI/HIVE diagnosis (*n* = 43). Based on these findings, recommendations are proposed in the latter part of the review.

### 3.4 Brain regions investigated

Several brain regions were investigated across all studies which included the adjacent cortex, anterior cingulate cortex (ACC), basal ganglia (BG), brain stem, caudate, caudate nucleus, cerebellum (CB), cerebral white matter (WM), cerebrum, choroid plexus, cingulate gyri, corpus callosum, cortex, dentate nucleus, deep white matter (DWM), entorhinal cortex, frontal cortex (FC), frontal lobe, frontal white matter (FWM), globus pallidus (GP), hippocampus, hypothalamus, insular cortex, internal capsule, medulla (MED), mid frontal cortex (MFC), middle frontal gyrus (MFG), midbrain (MB), neocortex, neostriatum, nucleus basalis of Meynert, occipital cortex (OC), occipital lobe, pallidum, parietal cortex, parietal lobe, pituitary, pons (PN), putamen, right dorsolateral prefrontal cortex, sensory cortex (SC), spinal cord (SPC), subcortical WM, temporal cortex (TC), temporal lobe, thalamus, thoracic and white matter. The majority of studies investigated the FC (*n* = 24), BG (*n* = 21), frontal lobe (*n* = 14), hippocampus (*n* = 10), CB (*n* = 5) and PN (*n* = 5). Three studies have not reported the brain regions that were investigated (Table 2).

**TABLE 2:** Studies reporting the association between HIV‐associated neuroinflammation and neurocognitive impairment/HIV encephalitis in PLWH.

References	Sampling technique	Brain section	Markers	Major findings
33	Enzyme‐linked immunosorbent assay (ELISA)	Frontal cortex (FC), caudate nucleus, insular cortex, basal ganglia (BG), thalamus, hypothalamus, hippocampus, superior cerebellum (CB), midbrain (MB), pons (PN) and medulla (MED)	Interleukin (IL)‐1, IL‐3, IL‐6, and tumour necrosis factor (TNF)‐α	All cytokine concentrations in the extracted tissues were uniformly low except for an occasional modest level of TNF‐α. Regional analysis of cytokines showed higher concentrations of TNF‐α in the BG of people living with HIV (PLWH) and HIV encephalitis (HIVE). Regional analysis of cytokines in cases with minimal HIVE showed a gradient of TNF‐α with the highest concentration in BG. A similar analysis in cases with severe HIVE showed a uniform elevation of TNF‐α in all three regions (cortical grey, cortical white, and deep grey matter).
34	Immunohistochemistry (IHC)	Temporal cortex (TC), BG, and brain stem	Major histocompatibility class II (MHC‐II) antigen, human Leucocyte antigen – DR isotype (HLA‐DR), IL‐1 and TNF‐α	Microglial activation was identified by higher levels of HLA‐DR, TNF‐α and IL‐1 expression. Severe microglial activation was only found in AIDS participants. Microglial activation was less pronounced but obvious in pre‐AIDS cases and weak or absent in seronegative patients. There was no clear distinction in the level of microglia activation between the HIVE/HIV‐associated dementia (HAD) compared to HIV non‐encephalitis (HIVnE) cases.
35	(IHC)	Cortex and white matter (WM)	TNF‐α, IL‐1α, IL‐4 and IL‐6	IL‐1α, IL‐4, IL‐6 and TNF‐α were higher in pre‐AIDS brains compared to HIV‐negative controls Neurocognitively impaired (NCI)/HIVE AIDS brains had lower levels of TNF‐α, and IL‐4 compared to pre‐AIDS brains. HIVE brains had higher levels of IL‐1α compared to pre‐AIDS brains. No differences were found for IL‐6 when comparing HIVE to pre‐AIDS brains.
36	Immunocytochemistry (ICC)	Frontal lobe	Glial fibrillary acidic protein (GFAP)	HIVE cases had significantly higher values of white and grey matter GFAP positivity, than all other case groups (all *p* < 0.05). The severity of NCI correlated positively with the number of GFAP‐positive astrocytes both for WM (Spearman rank order, RS = 0.537, *t* = 2.29, *p* = 0.02) and grey matter (RS = 0.633, *t* = 2.95, *p* < 0.006).
37	IHC	BG and hippocampus	Cluster of differentiation (CD)68, CD3, CD8, CD20, GFAP and MHC‐II	In the BG, CD68 levels were similar in highly active antiretroviral therapy (HAART)‐treated brains, AIDS, and pre‐symptomatic and HIVE brains however, all of these cases demonstrated higher levels compared to HIV‐negative controls (*p* = 0.004). No significant differences in BG CD68 levels were found between HIVE and AIDS/pre‐symptomatic brains. In the hippocampus, CD68 levels in HAART cases were similar to HIVE and significantly higher than those seen in AIDS, pre‐symptomatic, or control brains (*p* = 0.01). BG CD68 levels were higher in HIVE brains compared to AIDS, pre‐symptomatic, or control brains. HIVE cases show the highest levels of GFAP compared to all other groups; however, this was not significant. CD3, CD8, CD20 and MHC‐II were not significantly different between HIVE and HIVnE groups.
38	IHC	Frontal, temporal lobes and the thalamus	CD68	HIVE groups displayed more CD68‐positive microglia in the grey matter than the HIVnE groups (*p* < 0.001). No difference was detected between the HIVE and HIVnE groups in terms of CD68 positivity in the frontal lobe.
39	IHC and polymerase chain reaction (PCR)	FC	IL‐1β and IL‐10	IL‐1β gene expression was detected in the HIV‐associated dementia (HAD) group at significantly higher levels than in the group of neurocognitive normal (NCN) group (*p* < 0.025) and in the control group (*p* < 0.025). IL‐10 gene expression was also expressed at significantly higher levels in the HAD group compared with the control (*p* < 0.01) and NCN group (*p* < 0.01).
40	Western blot	Occipital lobes	Osteopontin (OPN)	OPN levels were higher in PLWH with more severe NCI compared to HIV‐infected individuals without or with mild NCI (*p* = 0.0387).
41	IHC	Hippocampus, putamen, and internal capsule, FC	HLA‐DR, GFAP, and ionised calcium‐binding adapter molecule (Iba)‐1	NCI was not associated with Iba‐1, GFAP or HLA‐DR in any of the investigated brain regions.
42	In situ hybridisation	FC, hippocampus, and brain stem	C‐C chemokine ligand (CCL)2	Cells expressing CCL2 RNA were often observed in perivascular regions. No CCL2‐positive cells were seen in normal brain tissue or tissue from patients without dementia and only found in brains with dementia.
43	Immunostaining, IHC and ICC	Cerebral WM and adjacent cortex and/or BG	CD14, CD45, and CD68	In HIV‐negative control brains, CD14 expression was limited to macrophages in the perivascular and meningeal locations and parenchymal microglia were CD14‐negative. In HIV‐seropositive control brains, a spectrum of parenchymal microglial CD14 expression was seen ranging from normal levels to those seen in HIVE cases. In HIVE cases, microglial CD14 expression was the most robust. CD45 expression was further upregulated in microglia and macrophages in HIVE. No data were reported for comparing CD68 levels between groups.
8	IHC	Frontal lobe	CD45, CD45RA, CRB, CD45RB, CD45RC, CD45RO, CD68, CD3, and CD8	αCD45RA and αRC‐reactive cells were rare averaging—one cell per 400× field in HIVE and even fewer in control brains. Neither CD45RA nor CD45RC counts were significantly different among the groups investigated. CD45RB parenchymal counts were uniformly high in all three groups (*p* > 0.05), HIV‐positive, HIV‐negative and HIVE. Perivascular CD45RB counts were significantly elevated in HIVE compared with HIV−negative (*p* < 0.001) or HIV‐positive (*p* < 0.01) groups. When CD45RO counts were compared between groups, the parenchymal (but not perivascular) CD45RO counts in HIVE were significantly elevated compared with the HIVnE group. The total CD45RO counts were also significantly elevated in HIVE compared with HIVnE.
44	Immunofluorescence	Mid‐frontal cortex (MFC), caudate nucleus, insular cortex, BG, thalamus, hippocampus, CB, MB, PN, MED, and spinal cord (SPC).	CD68 and HLA‐DR	HIV‐1 sections demonstrated a mild increase in CD68‐positive cells and cell processes within both the cortex and CB. No overall increase between HIVE and HIVnE of HLA‐DR expression was observed in any region compared to the same region in the control section. Compared to both HIV‐1 and control sections, HIVE sections demonstrated a marked increase in parenchymal and perivascular CD68‐positive cells, including CD68‐reactive microglial nodules, within the cortical WM, the deep grey matter, and, to a lesser extent, the cortical grey matter. Cerebellar sections, on the other hand, contained only a slightly greater number of immunoreactive cells than that observed in all HIV‐1 sections.
45	PCR and IHC	FC	IL‐1, IL‐6 and TNF‐α	IL‐1 transcription levels were significantly higher in PLWH, and latent HIV compared to HIVE (*p* < 0.05). Significant upregulation of IL‐6 transcript was observed in latent HIV cases compared to HIV and HIVE cases. TNF‐α transcript levels were not significantly different between groups. IL‐6 immunoreactivity was also higher in the astroglial cells in the latent and HIVE group compared to the HIV group.
46	IHC	Frontal lobe WM, FC and BG	Iba‐1	Iba1‐immunoreactive cells were higher in sections from subjects with NCI compared to the NCN groups (*p* < 0.05). Iba1 expression levels were found to be higher in the NCI group without HIVE.
47	PCR and ELISA	FC	Transforming growth factor (TGF)‐β1 and TGF‐β2	TGF‐β1 messenger (m)RNA levels were higher in NCI brains when compared to NCN brains and to nearly negligible amounts in control brain tissue (*p* < 0.05). TGF‐β2 mRNA was increased in NCI brains by 10‐fold (*p* < 0.01) compared to NCN brains and control brains. It is noteworthy that TGF‐β2 protein levels in NCI brain tissue were also significantly higher compared to the control group (*p* < 0.01).
48	PCR microarray	FC	All differential regulated genes due to HIVE	Genes involved in several categories were dysregulated in HIVE. Neuroinflammation genes were upregulated in HIVE compared to cognitively normal (CN) controls. Upregulation of interferon‐inducible genes in the HIVE with methamphetamine using group (and not HIVE alone), which together as a gene group was highly statistically significant (*p* = 0.0064).
49	IHC	FC	GFAP and Iba‐1	GFAP and Iba‐1 were increased brain tissue from aged HIVE patients compared to young HIVE patients. GFAP immunoreactivity and signal were elevated in young and aged HIVE tissues versus young and aged HIV positive samples; however, GFAP signal was most intense in tissues from aged HIVE patients. Iba‐1 signal intensity was increased in brain tissues of young and aged HIVE patients compared to HIV positive patients from both groups, with cells from HIVE tissues having more extended processes in greater numbers.
19	PCR	FC	TNF‐α	TNF‐α mRNA levels were significantly higher in the brains of Hispanics diagnosed with mild neurocognitive disorder (MND), compared to NCN controls (*p* < 0.05). Significantly higher levels of TNF‐α mRNA were found for the Hispanic group compared to the non‐Hispanic group (*p <* 0.01).
50	IHC	N/A	CD14, CD16, and HLA‐DR	Compared to HIV‐negative controls, there were increased numbers of perivascular CD14+ and CD16+ mononuclear phagocytes in HIVE brains. The majority of these cells identified in microglial nodules and the perivascular infiltrate were CD14+/CD16+. CD14+/CD16+ and HLA‐DR were higher in HIVE compared to HIVnE brains.
51	IHC	N/A	CD68, CD14 and CD16,	Higher levels of CD68 cells were found in HIVE brain tissue sections when compared to brain tissue from HIVnE (*p* = 0.016) and seronegative controls (*p* = 0.002). The total number of parenchymal CD68 cells was also significantly increased in HIVE brains. Increases in parenchymal macrophages in HIVE were observed with tissue sections stained for CD16, relative to HIV infected/HIVnE (*p* = 0.03) or normal controls (*p* = 0.005). CD14 levels were not significantly different between HIVE and HIVnE brains.
52	IHC	Cortex (mostly frontal), BG, and MB or PN	GFAP, and HLA‐DR	HIV infection without encephalopathy showed a clear increase in GFAP and HLA‐DR immunoreactivity when compared with normal tissues. GFAP and HLA‐DR were not significantly different between HIV and HIVnE groups.
53	IHC and PCR	MFG and the BG	HAM‐56	The mean summary score for HAM‐56 was significantly higher in the severely demented group compared with the non‐demented group (*p* = 0.04)
54	IHC	MFG, parietal cortex and CB	GFAP and HLA‐DR	HLA‐DR and GFAP were increased in the MFG and CB of NCI brains compared to NCN brains No differences for HLA‐DR were found in the parietal cortex.
55	PCR and IHC	FC	IL‐1β and IL‐33	IL‐1β (10‐fold) transcript levels were increased in the brains of PLWH with NCI and HIVE compared to the brains of NCN and HIV‐uninfected controls. No significant differences were found in IL‐33 transcript levels between the groups.
56	IHC	Frontal lobe and BG	TNF‐α and GFAP	TNF‐α ‐positive cell density was significantly high in the frontal cerebral cortex and BG in NCI brains compared with that in NCN brains (*p* < 0.01) and also with that HIV‐negative controls (*p* < 0.01). There was no statistical significance in the density of TNF‐α‐positive cells between NCN brains and HIV‐negative controls. The BG also showed a significant difference in the density of TNF‐α‐positive cells between NCI brains compared with that in NCN brains (*p* < 0.01) and also with that HIV‐negative controls (*p* < 0.01). The GFAP‐positive astrocyte density in NCI brains tended to be increased compared with NCN brains, but no significant difference between them was obtained.
57	ICC	MFC	C‐X‐C motif chemokine ligand (CXCL)12	In the MFC, CXCL12‐immunoreactive astroglia cells were most abundant in the subpial region and WM. In the astroglial cells, levels of immunoreactive CXCL12 were not significantly different among the four groups and did not correlate with the presence/absence of HIVE and/or neuronal damage (ND). In the brains of control cases, CXCL12 immunoreactivity was primarily localised to astrocytes and microglial cells whereas, in HIVE cases intense CXCL12 immunoreactivity was observed in microglia and neurons.
58	PCR and IHC	OC	Microtubule‐associated protein‐2 (MAP‐2), HLA‐DR and GFAP.	MAP2 was significantly correlated with global neurocognitive functioning and HIV‐associated neurocognitive disorder (HAND) severity. GFAP levels in the putamen were associated with motor, (*r* = 0.24, *p* = 0.034), and HLA‐DR in the FC with learning (*r* = 0.27, *p* = 0.017) outcomes. These correlations were not significant after correction using the FDR.
59	PCR and IHC	Right dorsolateral and MFC.	HLA‐DR, Iba‐1 and GFAP	None of the histopathological nor genetic markers besides MAP2 (at an FDR of 0.05) was associated with neurocognitive outcomes.
60	Cytokine labelling	MFC, cortical and subcortical regions	IL‐1β, IL‐2 and TGF‐β1	IL‐1β and TGF‐β1 were higher in cases with moderate HIVE in the FC compared to HIVnE. IL‐2 was not significantly different between HIVE and HIVnE brains.
61	PCR and IHC	FC	All differentially expressed genes, GFAP and CD45	In the HIVE brain, neuroinflammatory genes such as IgG heavy constant‐g3, major histocompatibility complex (MHC) classes 1A, C, F, h2‐ microglobulin and bone marrow stromal cell antigen‐2 (also known as B‐cell growth factor) were significantly upregulated. No significant differences in GFAP and CD45 between HIVE and HIVnE cases.
62	PCR	Frontal lobe	TNF‐α and IL‐1β	mRNA of both cytokines TNF‐α and IL‐1β showed significant up‐regulation in HAD brains compared with non‐dementia HIV/AIDS patients.
63	PCR	Brain and SPC tissue (cerebrum, CB, and brain stem)	TNF‐α, MIP‐1α and MIP‐1β	Expression of TNF‐α, MIP‐ 1α, and MIP‐ 1β strongly correlated with NCI and occurred in regions where HIV‐ 1‐infected cells were most plentiful and were localised primarily to the viral negative cells.
64	PCR and IHC	FC	CX3CL1	CX3CL1 mRNA levels were found to be significantly higher in the brains of NCI compared with the NCN and HIV‐negative controls (*p* < 0.05). CX3CL1 proteins levels were found to be overexpressed in the brains of NCI compared with the NCN. CX3CL1 protein immunoreactivity was found to be associated with astrocytes but not with neurons.
65	PCR	Cortex, BG, WM, and CB	TNF‐α, IL‐1β, IL‐6, and CD14	TNF‐α mRNA levels were higher in all brain tissue of PLWH than in controls. TNF‐α mRNA levels were the highest in HIVE brain tissue (*p* < 0.05). IL‐1β mRNA levels were higher in the brains of PLWH compared to negative controls but no significant differences were found between HIVE and NCN brains. IL‐6 mRNA levels were lower in HIVE brains compared to PLWH, however, not significantly. CD14 mRNA levels were uniformly higher in patients with HIVE than in controls (*p* < 0.05).
66	IHC	N/A	MIP‐1α, MIP‐1β, CCL2, and RANTES (CCL5), CD68, HLA‐DR and GFAP	Severe HIVE brains had pronounced infiltration of CD68‐positive MDM into the brain parenchyma compared to mild HIVE. Severe HIVE brains had strong positive immunostaining for HLA‐DR in white matter microglia compared to a lower level of HLA‐DR found in mild HIVE. GFAP was found in different areas but was especially prominent in zones most affected by MDM infiltration and microglial nodule formation in severe HIVE, whereas astrocyte reaction was less obvious in mild HIVE. All chemokines were higher in severe HIVE compared to milder HIVE. Chemokines were not detected in HIV‐negative brains. HIVE was associated with viral infection, microglial activation, monocyte‐derived macrophage (MDM) brain infiltration, astrogliosis, and *β*‐chemokine expression.
67	IHC	Hippocampus	GFAP and HLA‐DR (LN3)	The number of GFAP reactive astrocytes increased in the 2 AIDS groups and was greatest in the HIVE group (*p* < 0.05). The increase in GFAP reactive astrocytes for both NCN HIV and HIVE was greatest in the CA4 region where it was 4‐fold and 6‐fold greater than controls LN3‐positive microglia were rare or absent in controls and increased in AIDS brains.
68	IHC	Hippocampus	CD45RO and CD68	Parenchymal CD45RO+ and CD3+T lymphocytes were abundant in seven of the eight hippocampal regions with local HIVE but were rare or absent in the hippocampus of the NCN group and the HIVEnE. The higher mean number of hippocampal CD45RO + T‐ lymphocytes in the HIVE patient group (*n* = 10) than in the NCN group (*n* = 11) and the control group (*n* = 7), A highly significant increase in T lymphocytes in those hippocampal regions with HIVE inflammatory nodules (*p* < 0.001). CD68+ macrophages significantly increased in all three hippocampal regions of the HIVE group but not in the NCN group.
69	ICC	FC	GFAP, HLA‐DR, CD45 and CD68	GFAP immunostaining of subcortical WM revealed increased astrogliosis in tissue from demented and nondemented AIDS brains compared to HIV‐negative control and pre‐AIDS brains. There was a statistically significant increase in the mean number of GFAP‐positive cells detected in tissue from the two AIDS groups compared to the control group (*p* = 0.04). The increased numbers of immunodetectable astrocytes were accompanied by hypertrophy of the astrocyte cell body and elongation of cell processes in the AIDS groups compared to the control group. There were no significant differences in GFAP between demented versus non‐demented groups. Microglial hypertrophy and increased expression of LCA (CD45), EBM‐I 1 (CD68), and HLA‐DR were observed in HIV‐infected brains compared to controls. However, no differences were found between demented and non‐demented brains.
70	PCR and IHC	Frontal lobe, neocortex, WM, and neostriatum	Galectin (Gal)‐9	Gal‐9 gene expression was significantly higher only in the FWM brain region of HIVE donors compared to controls (*p* = 0.016). Although the intensity of Gal‐9 staining did not significantly differ among groups, an increase in the number of Gal‐9 positive cells in HIVnE samples as compared to uninfected controls was observed (*p* = 0.037).
71	Immunofluorescence	FC	CD40 and CD68	CD68‐positive cells were more in the perivascular spaces of HIVE compared to HIV‐negative control cases. No data were reported comparing HIVE compared to HIVnE brains. CD40 was expressed higher in HIVE compared to normal HIV brains and HIV‐negative control brains. Microglia in severe HIVE cases were positive for CD40 and no immunostaining was detected in HIV‐negative controls.
72	IHC	WM, BG (GP and the FC	TNF‐α	For TNF‐α, the degree of staining in the frontal deep WM and the BG correlated with the stage of AIDS dementia complex (*p* = 0.0001 and *p* = 0.0017 respectively).
73	IHC and PCR	(FC) with adjacent deep white matter (DWM) and (BG), including the globus pallidus and frontal region	CXCL12	A greater number of positive CXCL12 staining astrocytes tended to be observed in the BG of subjects with moderate/severe HIVE when compared to those with mild HIVE (*p* = 0.12) and HIV‐negative controls (unadjusted *p*‐value = 0.04). RNA levels of CXCL12 in the FC were compared and similar levels were detected in subjects with HIVE compared to those HIVnE. No difference in the number of CXCL12 ‐positive neurons was found in the FC between groups In HIV subjects with moderate to severe disease, the number of CXCL12 ‐positive neurons and astrocytes in the BG was increased compared to that in subjects with mild disease and controls
74	IHC	FC and BG	Tumour necrosis factor‐related apoptosis‐inducing ligand (TRAIL)	TRAIL was associated with mononuclear phagocytes (MP) (by colocalisation with HAM‐56) in both the FC and BG regions of HIVE brains. The percentage of macrophages expressing TRAIL in HIVE brain tissue (the ratio of dual HAM‐56‐ and TRAIL‐stained cells to total HAM‐56‐stained cells) was increased (69.4 ± 19% as compared to 52.9 ± 10%, *p* < 0.01) when compared to PLWH without neurological disease. Additionally, several of the macrophages expressing TRAIL were HIV‐1 infected as detected by co‐localization with HIV‐1 p24 antibodies).
75	PCR	N/A	TNF2 and HLA‐DR3	TNF2 and HLA‐DR3 did not associate with the presence of HIVE/leukoencephalopathy.
76	IHC	MFG, superior temporal and cingulate gyri, hippocampus, occipital cortex, nucleus basalis of Meynert, head of the caudate nucleus, pallidum, WM of the centrum ovale, dentate nucleus, SC at cervical, thoracic and lumbar levels	GFAP	GFAP‐positive astrocytes in the WM of the frontal lobe were higher in AIDS brains (*U* = 4, *p* = 0.0027) than in controls. AIDS patients with cognitive disorders did not differ significantly from others for GFAP.
77	IHC	Cortical WM (from the BG including the putamen and pallidum, and from the deep WM of the centrum ovale)	TNF‐α and GFAP	The density of TNF‐α positive cells was correlated with the mini‐mental state examination (MMSE) score in the deep WM (*p* = 0.022), and the BG and MFC (*p* = 0.037 for each). AIDS patients with myelin pallor (*n* = 5) had significantly higher numbers of TNF‐α‐positive cells in the deep‐white matter than other AIDS patients *(p* = 0.0045). A low density of TNF‐α positive cells, most of which were in close contact with the vessels, was found in normal controls and the density of TNF‐α positive cells was significantly higher in AIDS patients (*p* = 0.0012 for the deep WM; *p* = 0.0016 for the subcortical WM; *p* = 0.0007 for the MFC and BG). TNF‐α was significantly higher in HIVE (*n* = 3) compared to poliodystrophy (*n* = 6), in two regions: The deep WM and the MFC (*p* = 0.039; and *p* = 0.0201, respectively). No significant difference was found in the density of GFAP‐positive cells among the neuropathological subgroups of AIDS patients.
78	ICC	Occipital lobe	OPN, CD68, Iba‐1, and GFAP	Compared to the HIV‐negative group, OPN levels did not differ significantly compared to the groups (NCN, NCI). Not significant, but there was a trend of increased OPN in the NCI group. There were significantly higher levels of OPN between PLWH and the NCI groups compared to the amyotrophic lateral sclerosis (ALS) group (*p* = 0.011). CD68 was significantly lower in certain cases when comparing NCI brains to normal controls, however, the levels between NCN and NCI brains were not reported. GFAP were not significantly different between NCN and NCI brains. Iba‐1 levels normalised to OPN intensity were significantly lower in NCI brains compared to normal controls, however, the levels between NCN and NCI brains were not reported.
79	PCR and IHC	Frontal lobe and subcortical WM	Tumour necrosis factor receptor (TNFR)I, TNFRII and TNF‐α	The TNFRI and TNFRII gene expression were significantly higher in PLWH compared to HIV‐negative controls in the cerebral WM (*p* < 0. 01). TNFRI and TNFRII were not significantly different between HIVnE and HIVE brain tissue. TNF‐α immunoreactivity was significantly higher in the brain tissue of PLWH compared to HIV‐negative controls (*p* < 0.05). There were no significant differences in TNF‐α immunoreactivity between HIVnE and HIVE.
80	PCR and ELISA	FC and BG (CB, and WM)	IL‐1β, Matrix metalloproteinases (MMP)2 and tissue inhibitors of metalloproteinases (TIMP)	TIMP‐1 protein levels in brain tissue were significantly lower in PLWH and NCI PLWH when compared to controls (*p* < 0.001) MMP‐2 protein levels showed the opposite trend and were significantly higher in PLWH (*p* < 0.01) and NCI/HIVE brains (*p* < 0.0001). IL‐1β RNA was upregulated in PLWH and NCI PLWH as compared to seronegative controls (*p* < 0.001).
20	PCR	FC	IL‐1β	IL‐1β levels were significantly higher in all brains with NCI compared to NCN brains, despite ART regimens.
81	IHC	FWM and BG	CD16, CD163, CD68, HLA‐DR and GFAP	Significant accumulation of MΦs/microglia in HIVE and HIVnE, as compared to control tissues, revealed by CD68 immunopositivity. Significant CD16 expression was observed on MΦs/microglia that accumulated within the brain parenchyma, as well as those that comprise perivascular cuffs and nodular lesions, in the brains of patients with HIVE and was absent in seronegative brains. CD16 expression was also seen in the CNS of the HIVnE group although this was generally to a much lesser extent than that observed in HIVE. Significant accumulation of CD163+ MΦs and microglia were observed in the CNS of patients with HIVE within the parenchyma, perivascular cuffs, and nodular lesions. In normal brain, CD163+ expression was seen on perivascular MΦs, but not parenchymal microglia consistent with normal CD163 expression in the CNS. HIVnE subjects showed some accumulation of CD163+ perivascular MΦs but not to the same degree as HIVE. There were no significant differences between HIVnE and HIVE brains for CD68 and CD16. HIVE, HLA‐DR expression in the brains of subjects with none‐encephalitis was, for the most part, not observed on cells in the parenchyma, however, when present, parenchymal HLA‐DR was observed, it was seen on aggregates of cells, or what we've termed, ‘soft nodules’ because they appear as mild cell aggregates, rather than the distinct large nodular lesions seen in HIVE. In seronegative brains, HLA‐DR was predominantly limited to rare expression by perivascular MΦ. Increased frequency of GFAP + astrocytes was observed in WM of subjects with none‐encephalitis and HIVE, as compared to those without HIV, which corresponded with greater astrocyte hypertrophy. In cortical grey matter (GM), GFAP expression by astrocytes in areas away from blood vessels was only observed in HIVE.
82	IHC	Frontal lobe	GFAP	GFAP was not significantly different between HIVE and HIVnE groups.
83	IHC	Entorhinal cortex, hippocampus, subcortical WMFC and BG	GFAP	Mean GFAP values were significantly greater in the HAD entorhinal cortex, hippocampus and subcortical WM than those recorded in NCN controls, while differences in FC and BG proved negligible.
84	PCR	Cortex, subcortical, and deep WM from the right frontal lobe and GP from the BG	IL‐1β, IL‐6, IFN‐γ, TGF‐ β1, TGF‐ β2, TNF‐α, IL‐2, IL‐4, IL‐10 and monokine induced by gamma interferon (MIG)‐2	TGF‐β1 was easily detectable in most brains while amounts of TNF‐α, IL‐1β, and IL‐6 were more variable. TNF‐α was significantly higher in NCI AIDS patients, while IL‐1β tended to be lower. No differences between the NCI and NCN AIDS groups were detected for IFN‐γ, IL‐6, TGF‐β1, or TGF‐ β2. IL‐2, MIG‐2 and IL‐10 transcripts were undetectable in all brains. IL‐4 transcripts were undetectable in the brains of NCI PLWH but were detected in the brains of many CN and control subjects.
85	ICC	FC	GFAP	GFAP was significantly higher in HIVE compared to HIVnE brains.
86	IHC	Frontal lobe and PN	IL‐1β and TNF‐α	IL‐1β was detected only in the cells of the inflammatory lesions in all 11 cases with HIVE. TNF‐α was also detected in the inflammatory lesions of 7 cases with HIVE. Without HIVE, IL‐1β and TNF‐ *α* were detected in very few perivascular cells.
87	PCR	FC and BG	IL‐8	Expression levels of IL‐8 were 2.8‐fold higher in HIVE individuals compared with controls (*p* < 0.05) and 2.6‐fold higher compared with HIV‐1‐seropositive individuals (*p* < 0.05).
88	PCR	Caudate and ACC	CCL2, CCR5 and CXCR4	In the caudate and ACC: none of the marker's transcript levels were significantly different when comparing NCI and NCN PLWH. CCL2 transcript levels were significantly higher in PLWH in both caudate and ACC compared to HIV‐negative controls.
97	ICC	FC or BG (putamen)	IL‐1	IL‐1 immunoreactivity was higher in all cases of HIVE compared to HIVnE cases. IL‐1 staining in control brains was minimal. IL‐1 is strongly expressed in activated microglia and macrophages in HIVE.
90	IHC	Frontal lobe, Parietal lobe, temporal lobe occipital lobe, BG, hippocampus, CB, MB, PN, MED, thalamus, corpus callosum, pituitary, Mamlliary bodies, choroid plexus and putamen	CD8, CD68, S100 calcium‐binding protein MRP‐8 (S100A8)	CD8 was significantly higher in HAD compared to non‐dementia patients in the BG and parietal regions (*p* < 0.05). CD68 was significantly higher between HAD and NCN the difference was observed in BG, parietal and MB (*p* < 0.05). S‐100A8 immunohistochemical staining was also pronounced in the deeper midline and mesial temporal structure in HAD brains.

*Note*: *p* values were reported in this table for studies that reported the *p* values.

Abbreviations: ACC, anterior cingulate cortex; AIDS, acquired immunodeficiency syndrome; AM, amygdala; BG, Basal ganglia; CB, cerebellum; CCL, C‐C chemokine ligand; CCR, chemokine receptor; CD, Cluster of differentiation CXCL, Chemokine (C‐X‐C motif) ligand; CXCR, C‐X‐C chemokine receptor type; ELISA, Enzyme‐linked immunosorbent assay; FC, frontal cortex; FWM, frontal white matter; Gal, Galectin; GFAP, Glial fibrillary acidic protein GP, globus pallidus; HAART, Highly active antiretroviral therapy; HAD, HIV‐associated dementia; HIVE, HIVE encephalitis; HIVnE, HIV no encephalitis; HLA‐DR, Human Leucocyte Antigen – DR isotype (HLA‐DR); HO, Heme oxygenase; Iba‐1, ionised calcium‐binding adapter molecule; ICC, Immunocytochemistry; IHC, Immunohistochemistry; IL, Interleukin; MB, midbrain; MC, motor cortex; MED, medulla; MFC, Midfrontal cortex; MHC‐II, Major histocompatibility class II; MIP, macrophage inflammatory protein; MMP, Matrix metalloproteinases; MMSE, mini‐mental state examination; MND, mild neurocognitive disorder; MP, mononuclear phagocytes; mRNA, messenger RNA; NCI, neurocognitive(ly) impaired; NCN, cognitive/cognitively normal; ND, Neuronal damage; OC, occipital cortex; OPN, Osteopontin; PCC, posterior cingulate cortex; PCR, Polymerase chain reaction; PLWH, people living with HIV; PN, pons; RNA, ribonucleic acid; SC, sensory cortex; SPC, spinal cord; TC, temporal cortex; TGF, Transforming growth factor; TIMP, tissue inhibitors of metalloproteinases; TNF, Tumour necrosis factor; TNFR, Tumour necrosis factor receptor; WM, White matter.

### 3.5 Confounders: Viral load, CD4^+^ count and HIV‐1 subtype

We explored whether viral load, CD4^+^ count and HIV‐1 subtype influenced the associations between the level of neuroinflammation and NCI/HIVE as reported by studies investigating post‐mortem brain tissue. First, the majority of the studies (*n* = 41/61, 67%) have not reported data for antiretroviral therapy (ART) status. Of the *n* = 20/61 (33%) that have reported ART status, *n* = 15 studies reported that participants were receiving ART and *n* = 5 studies reported that participants were treatment naïve. From all selected studies, *n* = 18/61 (30%) have reported viral loads, with the majority (*n* = 17/18) reporting a detectable plasma/CSF viral load (>2.6 log copies/ml) in the respective studies. Only one study reported participants with viral suppression (<2.6 log copies/ml).^37^ A clear deduction could not be made due to only one study that included participants that were virally suppressed. From the non‐virally suppressed group, the results were not one‐sided as expected. A larger percentage of studies with non‐virally suppressed participants (*n* = 40/61, 66%) reported that inflammatory markers were significantly higher in NCI/HIVE groups compared to NCN groups (Supplementary Table S2). However, *n* = 21/61 (34%) of studies reported that certain inflammatory markers levels did not significantly differ between NCI/HIVE versus NCN/HIVnE groups.

Second, *n* = 26/61 (43%) have reported CD4^+^ count, and from these studies, all participants had CD4^+^ counts below 300 cells/μl. Two studies described that their cohorts had <300 cells/μl, however, participants were classified as having AIDS in these studies. Therefore, for this review, we have also stratified these participants as part of the <200 cells/μl (AIDS) group (Supplementary Table S3). When stratifying studies according to CD4^+^ count (<200 cells/μL (AIDS) or >200 cells/μL (non‐AIDS)), only one study had a mean cohort CD4+ count >200 cells/μL^69^ and this study reported that the inflammatory profile was associated with NCI/HIVE. The remaining *n* = 25/61 (41%) studies investigated cohorts with a mean/median CD4+ count of <200 cells/μL and 88% of these studies reported that the inflammatory profile was associated with HIVE/CI. Similar to the findings of viral load stratification, a clear deduction could not be made for CD4^+^ count due to only one study including participants that were stratified as having a mean cohort CD4^+^ count >200 cells/μl.

Last, none of the studies have sequenced HIV from these cohorts and therefore the HIV‐1 subtypes were not reported. However, geographical locations of these samples were provided for *n* = 47/61 (72%) of the included studies, with the majority of samples sourced from the United States of America (*n* = 32/61, 52%), followed by the United Kingdom (*n* = 6/61, 10%), Australia (*n* = 1/61, 2%), Vienna (*n* = 1/61, 2%), Argentina (*n* = 1/61, 2%), France (*n* = 2/61, 4%), Austria (*n* = 1/61, 2%). No commentary could be provided regarding the influence of subtype variation on the association of neuroinflammation and HAND/HIVE as reported in post‐mortem brain tissue.

### 3.6 Markers investigated

Several immune markers were investigated across the studies which included C‐C chemokine ligand (CCL)2, CCL5, chemokine receptor (CCR)5, cluster of differentiation (CD)3, CD8, CD14, CD16, CD163, CD20, CD40, CD45, CD45RA, CD45RB, CD45RC, CD45RO, CD68, CRB, C‐X‐C motif chemokine ligand (CXCL)1, CXCL12, CXCR4, Galectin (Gal)‐9, Glial fibrillary acidic protein (GFAP), HAM‐56, Human Leucocyte Antigen – DR isotype (HLA‐DR), HLA‐DR3, ionised calcium‐binding adapter molecule (Iba)‐1, Interferon (IFN)‐γ, Interleukin (IL)‐1, IL‐10, IL‐1α, IL‐1β, IL‐2, IL‐3, IL‐33, IL‐4, IL‐6, IL‐8, Matrix metalloproteinases (MMP)2, Microtubule associated protein‐2 (MAP‐2), macrophage inflammatory protein (MIP‐1)α, MIP‐1β, Monokine induced by gamma interferon (MIG)‐2, Osteopontin (OPN), S100 calcium‐binding protein MRP‐8 (S100A8), Transforming growth factor (TGF)‐ β1, TGF‐β2, Tissue inhibitors of metalloproteinases (TIMP), Tumour necrosis factor (TNF)2, TNF‐α, Tumour necrosis factor receptor (TNFR)I, TNFRII and Tumour necrosis factor‐related apoptosis‐inducing ligand (TRAIL). From these markers, GFAP (*n* = 19/61, 31%), TNF‐α (*n* = 14/61, 23%), CD68 (*n* = 12/61, 20%), HLA‐DR (*n* = 12/61, 20%), IL‐1β (*n* = 9/61, 15%), IL‐6 (*n* = 5/61, 8%), and Iba‐1 (*n* = 5/61, 8%) were the most commonly investigated markers. All of these markers highlighted above were either measured by their RNA gene expression (*n* = 11/61, 18%), protein expression (*n* = 37/61, 61%) or a combination of these two approaches (*n* = 13/61, 21%) (Table 2).

### 3.7 Markers associated with NCI/HIVE in PLWH

Across all studies, certain markers were associated with NCI and/or HIVE in PLWH. Gene‐specific transcript levels were assessed for their association with NCI/HIVE including the markers Bone marrow stromal cell antigen 2, CCL2, CCL3, CCR5, CX3CL1, CXCL12, CXCR1, CXCR4, CD14, Gal‐9, h2‐microglobulin, HLA‐DR3, IgG heavy constant‐g3, IL‐1, IL‐1β, IL‐2, IL‐4, IL‐6, IL‐8, IL‐10, IL‐33, Major histocompatibility complex (MHC) classes 1A, C, F, MIP‐ 1α, MIP‐ 1β, MIG‐2, TGF‐β1, TGF‐β2, TNF2, TNF‐α, TNFRI and TNFRII (Supplementary Table S4). Several gene transcripts were not associated with CI/HIVE (Supplementary Table S4). However, the gene transcripts that were associated with NCI and/or HIVE included higher expression levels of Bone marrow stromal cell antigen 2, CCL2, CX3CL1, CD14, Gal‐9, h2‐microglobulin, IgG heavy constant‐g3, IL‐1β, IL‐8, IL‐10, Major histocompatibility complex (MHC) classes 1A, C, F, MIP‐ 1α, MIP‐ 1β, TGF‐β1, TGF‐β2, TNF‐α and lower transcript expression levels of IL‐1 and IL‐6 (Supplementary Table S4).

The protein levels that were assessed for their associations with NCI/HIVE included inflammatory markers; CCL2, CD8, CD14, CD16, CD40, CD45, CD45RA, CD45RB, CD45RC, CD45R0, CD68, CX3CL1, CXCL12, Galectin‐9, GFAP, HAM‐56, HLA‐DR, Iba‐1, IL‐1, IL‐1α, IL‐1β, IL‐2, IL‐3, IL‐4, IL‐6, IL‐16, IL‐33, MAP2, MHC‐II, MIP‐ 1α, MIP‐1β, MMP‐2, OPN, RANTES, S‐100A8, TGF‐β1, TGF‐β2, TIMP‐1, TNF‐α, TNFRI. TNFRII and TRAIL (Supplementary Table S5). The inflammatory protein markers that were associated with NCI/HIVE included higher levels of CCL2, CD4, CD14, CD16, CD40, CD45, CD45RB, CD45RO, CD68, CXCL1, GFAP, HAM‐56, HLA‐DR, Iba‐1, IL‐1, IL‐1α, IL‐1β, IL‐6, IL‐16, MAP2, MIP‐ 1α, MIP‐1β, MMP‐2, OPN, RANTES, S‐100A8, TGF‐β1, TGF‐β2, and TRAIL and lower levels of IL‐4 and TIMP‐1, as well as higher and lower levels of TNF‐α (Table 2). Several inflammatory markers were also found not to be associated with NCI/HIVE (Supplementary Table S5).

**FIGURE 2:**
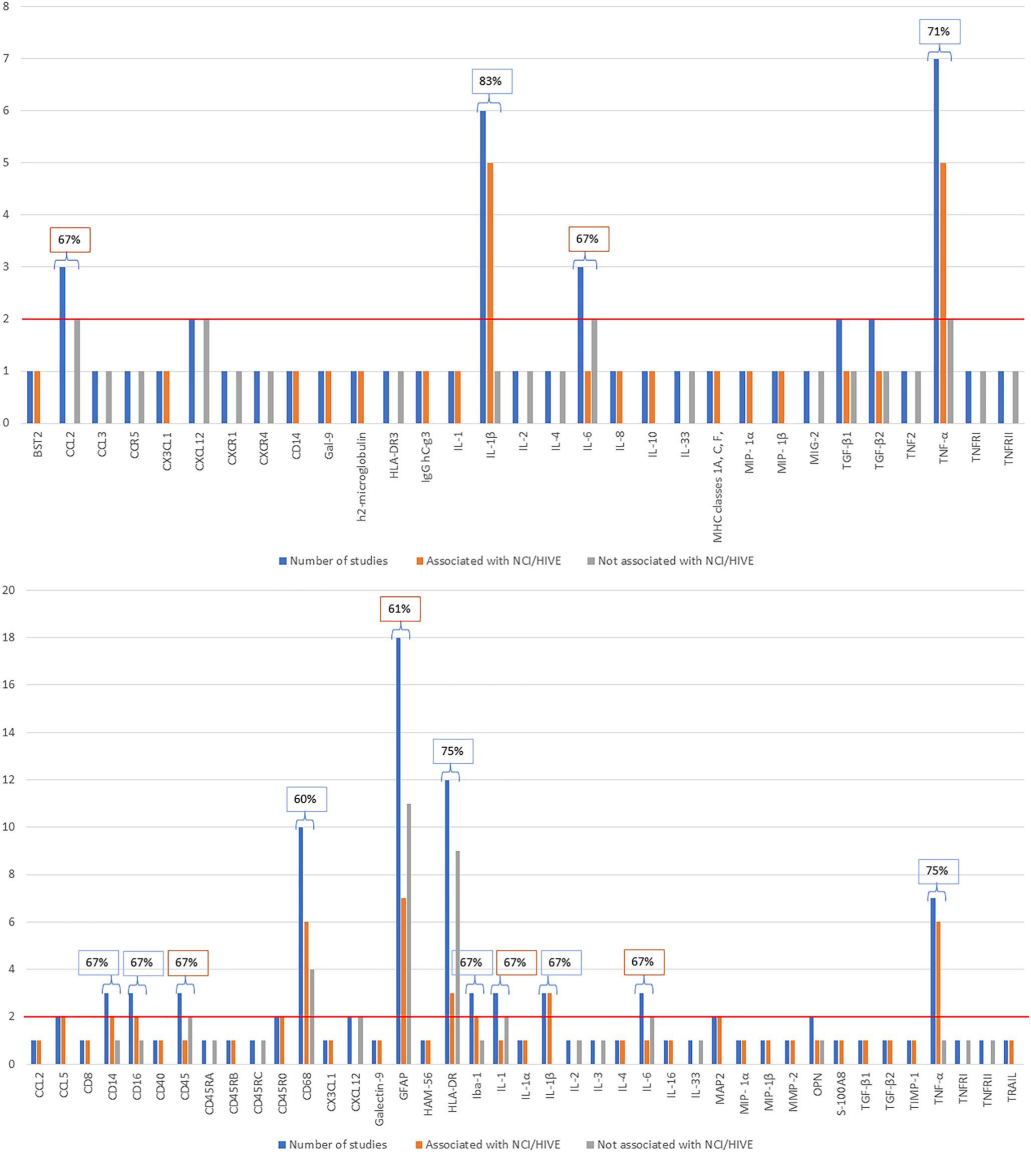
(a and b): The frequency of marker investigation across all studies. The blue bars indicate the number of studies which investigated a particular marker. Markers were considered noteworthy when at >2 independent studies investigated the marker: criteria one (red line cut‐off). The orange bars indicate the number of studies which reported significant associations of a particular marker with NCI/HIVE in PLWH. The grey bars indicate the number of studies which reported no significant associations of a particular marker with NCI/HIVE in PLWH. The percentage indicates the number of studies which report a relationship between a particular marker with NCI/HIVE in PLWH (orange or grey) in relation to the number of studies that investigated the marker (blue). The percentage >50% was indicated in boxes, with blue boxes indicating the association with NCI/HIVE, whereas orange boxes indicate no association with NCI/HIVE (criteria 2). (a) Gene expression markers and (b) Protein expression markers.

Certain markers were investigated more often and therefore would inherently have more supporting evidence. Therefore, we considered the frequency a marker was investigated when contextualising the findings. For this review, we applied a criterion for identifying ‘noteworthy’ markers as done previously.^17^, ^25^ For a marker (gene transcript/protein) to be noteworthy it had to be (1) investigated by three or more (>2) independent studies and (2) >50% of the studies investigating the marker had to report a consistent direction in their association with NCI/HIVE. In other words, if >50% of studies that investigated a particular marker found it to have the same direction in association with CI/HIVE, it was considered a noteworthy marker for future investigation. Transcript levels for CCL2, IL‐1β, IL‐6, and TNF‐α were investigated by > 2 independent studies, therefore meeting our first criteria (Figure 2a). Higher transcript expression levels of IL‐1β (*n* = 5/6, 83%) and TNF‐α (*n* = 5/7, 71%) were consistently associated with NCI/HIVE as reported in >50% or more of the studies that investigated these gene transcripts, therefore, meeting criteria two as a noteworthy marker (Figure 2a). Interestingly, CCL2 (*n* = 2/3, 67%) and IL‐6 (*n* = 2/3, 67%) were more commonly found not to be associated with NCI/HIVE (Figure 2a).

From the protein inflammatory markers, CD14, CD16, CD45, CD68, GFAP, HLA‐DR, Iba‐1, IL‐1, IL‐1β, IL‐6 and TNF‐α were investigated by > 2 independent studies, therefore meeting the first criteria for noteworthy (Figure 2b). Higher protein levels of CD14 (*n* = 2/3, 67%), CD16 (*n* = 2/3, 67%), CD68 (*n* = 6/10, 60%), Iba‐1 (*n* = 2/3, 67%), IL‐1β (*n* = 3/3, 100%) and TNF‐α (6/7, 85%) were consistently associated with NCI/HIVE as reported in >50% or more of the studies investigating these proteins, therefore, meeting criteria two as a noteworthy marker. From studies investigating TNF‐α, one found lower levels in NCI/HIVE whereas five studies found them to be increased and associated with NCI/HIVE (Supplementary Table S5). Interestingly, CD45 (*n* = 2/3, 67%), GFAP (*n* = 11/18, 61%), HLA‐DR (*n* = 9/12, 75%), IL‐1 (*n* = 2/3, 67%) and IL‐6 (*n* = 2/3, 67%) were more commonly found not to be associated with NCI/HIVE (Figure 2b). It is important to note that the inclusion of GFAP and HLA‐DR in a larger number of studies increases the likelihood of reporting non‐significant findings. Conversely, when only three studies focus on a single marker, the probability of discovering significant findings is increased. Therefore, when interpreting the findings presented in this review, it is essential to consider this contextual information.

Collectively, transcripts of IL‐1β and TNF‐α were more consistently associated with NCI/HIVE whereas transcripts of CCL2 and IL‐6 were more commonly not associated with NCI/HIVE. Proteins CD14, CD16, CD68, Iba‐1, IL‐1β and TNF‐α were more consistently associated with NCI/HIVE, whereas proteins CD45, GFAP, HLA‐DR, IL‐1 and IL‐6 were more commonly not associated with NCI/HIVE. Across all studies (gene transcripts and protein), IL‐1β and TNF‐α were associated with NCI/HIVE and IL‐6 was not associated with NCI/HIVE.

## 4 DISCUSSION

Several findings from studies on human post‐mortem brain tissue are highlighted in this review. These findings include the following: (1) The gene transcripts for IL‐1β and TNF‐α were consistently associated with NCI/HIVE, whereas the gene transcripts for CCL2 and IL‐6 were more consistently not associated with NCI/HIVE. (2) The protein expression levels of CD14, CD16, CD68, Iba‐1, IL‐1β and TNF‐α were more consistently associated with NCI/HIVE, whereas the proteins CD45, GFAP, HLA‐DR, IL‐1 and IL‐6 were more consistently not associated with NCI/HIVE. (3) On both a transcript and protein level, IL‐1β and TNF‐α were consistently associated with NCI/HIVE, whereas IL‐6 was not associated with NCI/HIVE. Based on the findings reported in this review, these markers were considered noteworthy for further investigation into their functions in the pathophysiology of NCI/HIVE, as well as their potential diagnostic, prognostic, and therapeutic potential.

Firstly, the transcript expression of TNF‐α and IL‐1β levels in the brain were consistently associated with the presentation of NCI/HIVE. These findings suggest that transcriptional inflammatory pathways for TNF‐α and IL‐1β may be active in the presence of HIV‐1 within the brain. HIV‐1, particularly its viral proteins, are able to activate several pathways to induce TNF‐α expression. For instance, (1) HIV‐1 gp120‐induced TNF‐α production by primary human macrophages is mediated by phosphatidylinositol‐3 (PI‐3) kinase and mitogen‐activated protein (MAP) kinase pathways,^98^, ^99^ (2) Viral protein R (Vpr) induced TNF‐α production by astrocytes are produced by Sur1‐Trpm4 channels^100^, ^101^ and (3) Tat induced mRNA TNF‐α induction is mediated by NF‐κB‐dependent pathways and is also linked to phospholipase C activation in macrophage and astrocytes.^100^ Of note, TNF‐α exerts differential effects in the CNS via its TNFR1 and TNFR2. TNFR1 predominantly activates pro‐inflammatory pathways and cell death, whereas activation of TNFR2 leads to neuroprotective signalling pathways.^102^, ^103^ Thus, future investigations on the balance of TNFR1 and TNFR2 expression levels are needed to elucidate the functional roles of elevated TNF‐α in the pathophysiology of NCI/HIVE.

Similarly, based on the presented findings, it can be suggested that IL‐1β mediated pathways may also be important in the development of HAND. HIV‐1 viral proteins can also activate pathways related to the expression IL‐1β. Examples of these include: (1) HIV‐1 gp120‐induced IL‐1β release by macrophages is mediated through CCR5 and coupling of G_i_α protein; concomitant activation of Lyn, PI3K and Pyk2; and the co‐ordinately acting of these kinases through the formation of a multi‐kinase signalling complex.^104^ (2) Vpr induced IL‐1β production occurs through the activation of p38 and stress‐activated protein kinase (SAPK)/c‐Jun N‐terminal kinase (JNK) in monocyte‐derived macrophages. (3) Tat induced IL‐1β production from human monocytes involves the phospholipase C/protein kinase C signalling cascade.^105^ Therefore, targeting signalling pathways linked to the induction of TNF‐α and IL‐1β could potentially reduce the inflammatory burden within the brain and mitigate potential neuronal damage.

Interestingly, we report that the transcript levels of CCL2 and IL‐6 were more consistently found not to be associated with NCI/HIVE. CCL2 is a widely investigated immune chemoattractant, and several studies have indicated that higher peripheral^106^, ^107^, ^108^ and CSF^109^, ^110^, ^111^, ^112^ levels were associated with NCI. Studies also reported inconsistent directions for the association of CCL2 with NCI, with higher peripheral and CNS levels^106^, ^108^, ^109^ and lower peripheral and CNS levels^107^, ^113^ in NCI. Therefore, the exact role of CCL2 in the development of HAND remains elusive. It has been argued the resultant neuronal damage by CCL2 presence is linked to its ability to recruit HIV‐1‐infected monocyte across the blood‐brain barrier.^114^, ^115^ It has also been suggested that CCL2 may be required for initiation but not the persistence of HIV infection‐mediated neurocognitive disease. A recent preprint study conducted by Kim and Colleagues suggested that CCL2 is required for the development of HAND during systemic EcoHIV infection of mice by promoting monocyte migration during the initial stages of infection, however, once HAND is established CCL2 is dispensable.^115^ Findings from this review support the notion that CCL2 levels in the brain may not be directly correlated with the presence of NCI/HIVE, however, may rather function as an initiator of NCI by monocyte recruitment.

Similarly, previous studies investigating peripheral, and CNS IL‐6 have reported inconsistent associations with NCI.^106^, ^107^, ^116^, ^117^ In this study, we report that IL‐6 was more consistently not associated with NCI/HIVE. It is important to note that inflammatory factors are influenced by several variables. Specifically, IL‐6 is influenced by older age, nonblack race, higher body mass index, lower serum lipid levels, HIV replication, low nadir CD4⁺ cell count, protease inhibitor use, comorbid conditions, and decreased estimated glomerular filtration rate (eGFR) during HIV‐1 infection.^118^, ^119^ Considering that participants across all studies were not well matched in terms of clinical variables, this may have contributed to inconsistent associations reported for IL‐6.

Secondly, this review suggests that CD14, CD16, CD68, Iba‐1, IL‐1β and TNF‐α proteins within the brain were consistently associated with NCI/HIVE. Several studies have implicated CD14, CD16, and CD68 as indicators and contributors to the development of HAND.^116^, ^120^, ^121^, ^122^, ^123^ The findings reported in this review support the notion that monocytes and macrophages play an important function in the mechanistic pathways leading to the development of HAND in the modern ART era. Further, Iba‐1, also known as Allograft inflammatory factor 1 (AIF‐1), is one of the most widely investigated protein markers of microglia activation. Similarly, across all studies reviewed, Iba‐1 was one of the more commonly investigated markers that showed a consistent association with NCI/HIVE. This is in contrast to other commonly investigated markers including GFAP, HLA‐DR, IL‐1 and IL‐6.

GFAP and HLA‐DR were amongst the most commonly investigated markers across all studies reviewed here, however, the majority of studies did not find GFAP and HLA‐DR to be associated with NCI/HIVE. GFAP is the major structural protein of astrocytes, and higher levels of this protein have been implicated in neurodegenerative disorders characterised by astrogliosis including Alzheimer's disease,^124^, ^125^ multiple sclerosis ^126^, ^127^ and cerebral vasculitis.^128^ Considering that one of the neuropathological hallmarks of HIV‐1‐associated dementia (HAD) is the proliferation of astrogliosis, it is expected that a strong association between higher GFAP and NCI/HIVE should exist. However, an earlier study by Sporer and colleagues has shown that CSF GFAP levels and the frequency of increased GFAP levels did not significantly differ between HIV‐infected patients with and without HAD, and, additionally, of HIV‐infected patients with opportunistic CNS diseases.^129^ The findings from human post‐mortem brain tissues on GFAP may not reflect the functions of astrocytes in the pathogenesis of NCI/HIVE. Evidence from fundamental research has showed that viral proteins that is, Tat protein, can activate astrocytes, causing glutamate imbalance that causes toxic glutamate signalling in neurons.^130^, ^131^ The HIV Nef protein on the other hand can downregulate GFAP expression, but still affect glutamate metabolism in astrocytes.^132^ It is reasonable to speculate that HIV‐associated proteins influence GFAP expression levels, explaining the non‐significant findings in the reported studies. Together the studies from post‐mortem human brain tissue reviewed here, as well as findings from animal models and cell cultures suggest that GFAP may not be a suitable indicator for the effects of astrocytes in the pathogenesis of NCI/HIVE in PLWH.

Here, we report that HLA‐DR was more commonly not associated with NCI/HIVE. Limited studies have investigated the association between HLA‐DR and NCI/HIVE. Among the limited studies on this topic, one study reported an association between HLA DR‐04 and NCI in participants from China. However, this was performed using HLA typing on blood samples.^133^ Another study that evaluated CSF HLA levels did not show an association with NCI.^134^ As shown in this review, studies using IHC and/or ICC to investigate brain tissue, more commonly demonstrated no association of HLA‐DR with NCI/HIVE. This difference in findings may be attributed to different techniques and biological specimen types. However, there is a need for further investigation into the role of HLA‐DR in the development of HAND.

Thirdly, we reported that on both a transcript and protein level, IL‐1β and TNF‐α were consistently associated with NCI/HIVE and IL‐6 was not associated with NCI/HIVE. This may suggest that pathways related to the expression of IL‐1β and TNF‐α may be fundamental in the development of NCI/HIVE. To our best knowledge, this is the first study to systematically review neuroinflammation and NCI/HIVE from studies using post‐mortem brain tissue. Therefore, these markers should be investigated to determine their diagnostics, prognostic, and therapeutic potential in addressing the persistent development of HAND in the modern ART era.

## 5 LIMITATIONS AND RECOMMENDATIONS

Limitations are evident in this review, and accordingly, several recommendations can be made. Firstly, several factors may have influenced the findings that were reported in the studies reviewed here and this made the interpretation of the findings more challenging. These factors included ART status, viral load/CD4^+^ count, age, gender, neuropsychological and neuropathological assessment, brain region investigated, and the HIV‐1 subtype. First, only *n* = 15/61 (25%) of the included studies have reported the use of ART, with *n* = 5/61 (8%) of studies reporting that participants were treatment naïve. The ART status for *n* = 37/61 (61%) of all included studies was not reported. Therefore, it is not clear what treatment status the included studies and findings present, making it challenging to interpret the findings for the association of neuroinflammation and neurocognitive impairment/HIVE. Furthermore, *n* = 46/61 (75%) of the studies were conducted prior to the year 2010, and the majority of studies classified as non‐virally suppressed may suggest a greater risk of participants having high levels of viraemia. These factors need to be taken into consideration when contextualising the findings that are reported in this review. Future studies should report the ART treatment status, duration of treatment and the exact treatment regimen, as these may have different CNS penetration effectiveness scores and may influence the viral and neuroinflammatory load and subsequent neuropathology (Letendre et al. 2008).

Secondly, a slightly higher number of studies (*n* = 26/61, 43%) have reported the mean/median CD4^+^ count of participants, and the majority of these studies indicating that participants had AIDS (<200 cells/μl). Therefore, this again highlights those participants in the reviewed studies were at an advanced stage of the disease and therefore may inherently have had a greater inflammatory response in the brain. This is supported by previous studies showing associations of higher TNF‐α in blood and CSF^135^, ^136^ and IL‐1β in CSF^136^ with lower CD4^+^ counts in PLWH.

Thirdly, the participants included in the reviewed studies had an age range of 21‐ to 56‐ years old. However, most studies did not investigate the influence of age on brain inflammation and therefore no conclusions could be drawn. Additionally, the majority of studies investigated male participants, and it is not clear if the reported levels of neuroinflammation in the brain were influenced by sex. It has been established that sex‐related differences can influence inflammatory levels, with men exhibiting higher plasma inflammation compared to women^137^ and men showing more associations between CSF inflammatory markers and cognitive performance compared to women with HIV.^119^ Consequently, no conclusions could be made regarding age and sex on the reported profiles. Future studies should aim to stratify and analyse neuroinflammatory profiles according to age and gender to fully contextualise the findings. Furthermore, all of the reviewed studies included adult individuals. Future studies should consider investigations using brain tissues of paediatric HIV and HIV‐exposed uninfected children.

Fourthly, we opted to examine the association of inflammatory markers with HIVE and NCI/HAND collectively rather than separately. This decision was influenced by the limited number of studies available per category when stratified. Furthermore, even if studies were stratified per category, it is relevant to note that the different studies employed varied approaches to evaluate neurocognitive impairment and neuropathology. It is known that different criteria for classifying HAND such as MSK, AAN, Frascati criteria,^138^, ^139^ vary in their strictness. The utilization of different criteria in assessing NCI and HAND across studies may have influenced the reported status of NCI. Recently Nightingale and colleagues highlighted criticisms of the HAND criteria, pointing out that (1) the statistical methods used for cognitive data analysis have the potential for a high false classification rate, (2) cognitive performance is strongly influenced by additional factors (e.g. complex educational, cultural, and socioeconomic factors), which may not necessarily correlate with the pathological state, and (3) cognitive impairment in individuals with HIV is often multifactorial and not solely attributable to the direct effect of HIV on the brain.^140^ In this regard, the studies that were included in this review employed different criteria for classifying HIVE neuropathology, ranging from single criterion (the presence of multinucleated giant cells (MGCs)) to multiple criteria (including multinucleated‐cell encephalitis, gliosis, white matter pallor, and vacuolar myelopathy in addition to other pathologies). The reported findings in this review should be interpreted in light of the variations in classification criteria, as these differences may have influenced the outcomes. This further highlights the need that future studies should develop standardized protocols for classifying HAND and HIVE, which would enhance comparability across studies.

Fifthly, the studies included in this review examined different brain regions, all of which may not have been equally vulnerable to HIV‐1‐induced neuroinflammatory effects. Previous investigations of post‐mortem brain tissue have demonstrated distinct patterns of higher type I interferon (IFN)‐stimulated transcript expression in specific brain regions, such as the posterior cingulate cortex, globus pallidus, and cerebellum.^141^ Additionally, we observed associations between certain markers and NCI/HIVE in particular brain regions. For example, HLA‐DR and GFAP were found to be increased in the MFG and CB of NCI brains compared to NCN brains. However, no differences were found when comparing these same markers in the PC between the groups.^54^

Lastly, it is known that the inflammatory response to HIV‐1 may be influenced by the HIV‐1 subtype, as shown in several fundamental studies.^142^, ^143^ None of the studies included in this review reported the specific HIV‐1 subtype, although the majority of studies were conducted on participants from the United States of America (*n* = 32/61, 52%), which is geographically related to subtype B.^144^ Therefore, these findings may reflect the inflammatory profile within the brains of participants with subtype B infection, rather than other geographical subtypes. Considering the significant impact that subtype variance has on the underlying mechanisms of HAND,^92^, ^145^ as well as clinical neurocognitive impairment in PLWH,^94^, ^146^, ^147^ we suggest that viral subtyping should be included as part of routine analysis in human post‐mortem studies of HIV.

## 6 CONCLUSION

Here, we conducted a systematic review of all studies investigating the relationship between neuroinflammation and NCI/HIVE using post‐mortem brain tissue. The main findings were: (1) In terms of inflammatory transcript expression, IL‐1β and TNF‐α showed a more consisted association with NCI/HIVE, while CCL2 and IL‐6 were more commonly not associated with NCI/HIVE. (2) Protein expression of CD14, CD16, CD68, Iba‐1, IL‐1β and TNF‐α demonstrated a more consistent association with NCI/HIVE, whereas CD45, GFAP, HLA‐DR, IL‐1 and IL‐6 were more commonly not associated with NCI/HIVE. (3) Both at the transcript and protein expression levels within the brain, IL‐1β and TNF‐α were consistently associated with NCI/HIVE, whereas IL‐6 consistently showed no association with NCI/HIVE. These findings highlight the commonly investigated markers in this line of research and emphasise their potential involvement in the development of NCI/HIVE. These markers and associated pathways should be considered for future studies aimed at developing improved therapeutics and diagnostics for NCI/HIVE.

## AUTHOR CONTRIBUTIONS

Monray E. Williams and Petrus J.W. Naudé analysed all data and wrote the manuscript. All authors read and approved the final manuscript.

## CONFLICT OF INTEREST STATEMENT

The authors declare that they have no competing interests.

## ETHICS STATEMENT

Not applicable.

## CONSENT FOR PUBLICATION

Not applicable.

## Supporting information

Supporting Information S1

Table S1

Table S2

Table S3

Table S4

Table S5

## Data Availability

All data generated or analyzed during this study are included in this published article [and its supplementary information files].
