# Supplementary material for: The relationship between HIV‐1 neuroinflammation, neurocognitive impairment and encephalitis pathology: A systematic review of studies investigating post‐mortem brain tissue
Source: Rev Med Virol. 2024 Jan 27;34(1):e2519. doi: 10.1002/rmv.2519 (PMC10909494; doi:10.1002/rmv.2519)
Supplement: Supplementary file 1 — Supporting Information S1 [file RMV-34-e2519-s005.docx]

**Pubmed (n = 1083) 24 May 2023**

(HIV [mh] OR HIV [tw] OR Acquired Immunodeficiency Syndrome [mh] OR “Acquired Immunodeficiency Syndrome” [tw] OR AIDS [tw]) AND (HIV associated neurocognitive disorders [mh] OR HAND [tw] OR neurocognitive [tw] OR cogniti* [tw] OR Executive Function [mh] OR executive [tw] OR Memory [mh] OR memory [tw] OR Attention [mh] OR attention [tw] OR Neuropsychological Tests [mh] OR AIDS Dementia Complex [mh] ) AND (Cytokines [mh] OR cytokin*[tw] OR Chemokines [mh] OR chemokine [tw] OR Inflammation [mh] OR inflammation [tw] OR Neurogenic Inflammation [mh] OR neuro-inflammation [tw] OR TNF [tw] OR Interleukins [mh] OR interleukins [tw] OR Microglia [mh] OR microglia [tw] OR Monocytes [mh] OR monocyte* [tw] OR sCD163 [tw] OR sCD14 [tw] OR sCD40 [tw] OR CD68 [tw] OR Neopterin [mh] OR Interferons [mh] OR Ionized calcium binding adaptor molecule 1 [tw] OR IBA1 [tw] OR Glial Fibrillary Acidic Protein [tw] OR S100 Calcium Binding Protein beta Subunit [mh] OR CHI3L1 protein, human [mh]) AND (post-mortem brain tissue [tw] OR postmortem brain OR Brain [mh] OR Immunohistochemistry [mh])

**Scopus (n = 69) 24 May 2023**

(hiv-associated neurocognitive disorders OR hand OR neurocognitive OR cogniti* OR neuropsychological tests OR AIDS dementia complex) AND (post-mortem brain tissue OR postmortem brain OR tissue OR immunohistochemistry) AND (cytokines OR cytokin* OR chemokines OR inflammation OR neurogenic inflammation OR neuroinflammation OR tnf OR interleukins OR microglia OR monocytes OR monocyte*)

**Web of science (n = 587) 24 May 2023**

TS=(HIV OR Acquired Immunodeficiency Syndrome OR" Acquired Immunodeficiency Syndrome" OR AIDS) AND TS=(HIV associated neurocognitive disorders OR HAND OR neurocognitive OR cogniti* OR Executive Function OR executive OR Memory OR memory OR Attention OR attention OR Neuropsychological Tests OR AIDS dementia complex) AND TS=(Cytokines OR cytokin* OR Chemokines OR chemokine OR Inflammation OR inflammation OR Neurogenic Inflammation OR neuroinflammation OR TNF OR Interleukins OR interleukins) AND TS=(Microglia OR microglia OR Monocytes OR monocyte* OR sCD163 OR sCD14 OR scd40l OR CD68 OR Ionized calcium binding adaptor molecule 1 OR IBA1 OR Glial Fibrillary Acidic Protein OR S100 Calcium Binding Protein beta Subunit OR CHI3L1 OR neopterin OR interferons) AND TS=(post-mortem brain tissue or brain or tissue or immunohistochemistry)

**Total: 1739**
