# Supplementary material for: The relationship between HIV‐1 neuroinflammation, neurocognitive impairment and encephalitis pathology: A systematic review of studies investigating post‐mortem brain tissue
Source: Rev Med Virol. 2024 Jan 27;34(1):e2519. doi: 10.1002/rmv.2519 (PMC10909494; doi:10.1002/rmv.2519)
Supplement: Supplementary file 2 — Table S1 [file RMV-34-e2519-s002.docx]

**Supplementary table 1**: Quality assessment of studies

| **Reference** | **Question 1** | **Question 2** | **Question 3** | **Rating** | |
| --- | --- | --- | --- | --- | --- |
| [78] | 1 | 1 | 0 | 2 | Low |
| [79] | 1 | 0 | 2 | 3 | Intermediate |
| [80] | 0 | 0 | 2 | 2 | Low |
| [81] | 1 | 2 | 0 | 3 | Intermediate |
| [29] | 1 | 2 | 2 | 5 | Intermediate |
| [82] | 2 | 0 | 2 | 4 | Intermediate |
| [83] | 1 | 1 | 2 | 4 | Intermediate |
| [84] | 0 | 1 | 2 | 3 | Intermediate |
| [85] | 2 | 1 | 2 | 5 | Intermediate |
| [86] | 0 | 0 | 2 | 2 | Low |
| [87] | 1 | 0 | 2 | 3 | Intermediate |
| [88] | 1 | 1 | 2 | 4 | Intermediate |
| [89] | 1 | 0 | 0 | 1 | Low |
| [90] | 2 | 2 | 2 | 6 | High |
| [91] | 1 | 0 | 0 | 1 | Low |
| [92] | 0 | 0 | 0 | 0 | Low |
| [93] | 1 | 2 | 0 | 3 | Intermediate |
| [94] | 1 | 2 | 2 | 5 | Intermediate |
| [13] | 1 | 2 | 2 | 5 | Intermediate |
| [95] | 1 | 0 | 0 | 1 | Low |
| [96] | 1 | 0 | 0 | 1 | Low |
| [97] | 1 | 1 | 1 | 3 | Intermediate |
| [98] | 1 | 0 | 2 | 3 | Intermediate |
| [71] | 1 | 0 | 2 | 3 | Intermediate |
| [99] | 0 | 0 | 0 | 0 | Low |
| [100] | 1 | 1 | 2 | 4 | Intermediate |
| [101] | 1 | 1 | 2 | 4 | Intermediate |
| [102] | 2 | 2 | 2 | 6 | High |
| [103] | 2 | 2 | 2 | 6 | High |
| [104] | 0 | 1 | 2 | 3 | Intermediate |
| [105] | 2 | 1 | 2 | 5 | Intermediate |
| [106] | 0 | 1 | 0 | 1 | Low |
| [107] | 1 | 1 | 0 | 2 | Low |
| [108] | 1 | 1 | 2 | 4 | Intermediate |
| [109] | 1 | 1 | 2 | 4 | Intermediate |
| [110] | 1 | 1 | 2 | 4 | Intermediate |
| [111] | 1 | 1 | 2 | 4 | Intermediate |
| [112] | 1 | 1 | 2 | 4 | Intermediate |
| [30] | 1 | 1 | 2 | 4 | Intermediate |
| *[113] | 1 | 1 | 0 | 2 | Low |
| [114] | 1 | 1 | 2 | 4 | Intermediate |
| [115] | 1 | 0 | 2 | 3 | Intermediate |
| [116] | 1 | 0 | 2 | 3 | Intermediate |
| [117] | 1 | 1 | 0 | 2 | Low |
| [118] | 1 | 0 | 0 | 1 | Low |
| [119] | 1 | 1 | 2 | 4 | Intermediate |
| [120] | 1 | 1 | 2 | 4 | Intermediate |
| [121] | 2 | 2 | 2 | 6 | High |
| [122] | 1 | 1 | 2 | 4 | Intermediate |
| [123] | 0 | 0 | 0 | 0 | Low |
| [14] | 1 | 2 | 2 | 5 | Intermediate |
| [124] | 1 | 2 | 2 | 5 | Intermediate |
| [125] | 1 | 0 | 0 | 1 | Low |
| [126] | 1 | 2 | 2 | 5 | Intermediate |
| [127] | 1 | 1 | 2 | 4 | Intermediate |
| [128] | 0 | 0 | 2 | 2 | Low |
| [129] | 1 | 0 | 2 | 3 | Intermediate |
| [130] | 0 | 0 | 0 | 0 | Low |
| [131] | 1 | 1 | 2 | 4 | Intermediate |
| [132] | 1 | 1 | 2 | 4 | Intermediate |
| [133] | 1 | 1 | 2 | 4 | Intermediate |

Questions were as follow: (1) Confounders: Did the study report on potential confounders (e.g., substance misuse, comorbid conditions (e.g., Hepatitis C (HCV)), neurological conditions and psychiatric disorders), a relevant exclusionary criterion and were these controlled for upon statistical analysis? (2) Study characteristics: Did the study report all key cohort information to contextualise the reported findings (i.e., age of participants at time of death, antemortem CD4+ count/viral load, use and duration of ART use)? And (3) HAND/ HIVE diagnosis: Did the study report the antemortem HAND diagnosis criteria and/or did the study clearly define the criteria for HIVE post-mortem?
