# Supplementary material for: The relationship between HIV‐1 neuroinflammation, neurocognitive impairment and encephalitis pathology: A systematic review of studies investigating post‐mortem brain tissue
Source: Rev Med Virol. 2024 Jan 27;34(1):e2519. doi: 10.1002/rmv.2519 (PMC10909494; doi:10.1002/rmv.2519)
Supplement: Supplementary file 3 — Table S2 [file RMV-34-e2519-s003.docx]

**Supplementary Table 2:** Studies reporting associations between neuroinflammation and neurocognitive impairment/HIV encephalitis when stratified according to viral suppression

| **Reference** | **Virally suppressed** | **Non-virally suppressed** | **Association of inflammatory markers with NCI/HIVE** | |
| --- | --- | --- | --- | --- |
|  |  |  | **Yes** | **No** |
| [81] |  | X | X |  |
| [29] | X |  | X |  |
| [85] |  | X |  | X |
| [90] |  | X | X |  |
| [93] |  | X | X |  |
| [94] |  | X | X |  |
| [13] |  | X | X |  |
| [102] |  | X |  | X |
| [103] |  | X |  | X |
| [105] |  | X | X |  |
| [113] |  | X |  |  |
| [114] |  | X | X |  |
| [121] |  | X |  | X |
| [14] |  | X | X |  |
| [124] |  | X | X |  |
| [126] |  | X | X |  |
| [131] |  | X |  | X |
| [133] |  | X | X |  |

**Abbreviations:** HIVE: HIV encephalitis; NCI: Neurocognitive impairment
