# Supplementary material for: The relationship between HIV‐1 neuroinflammation, neurocognitive impairment and encephalitis pathology: A systematic review of studies investigating post‐mortem brain tissue
Source: Rev Med Virol. 2024 Jan 27;34(1):e2519. doi: 10.1002/rmv.2519 (PMC10909494; doi:10.1002/rmv.2519)
Supplement: Supplementary file 4 — Table S3 [file RMV-34-e2519-s001.docx]

**Supplementary Table 3:** Studies reporting associations between neuroinflammation and neurocognitive impairment/HIV encephalitis when stratified according to CD4^+^ count

| **Reference** | **CD4^+^ <200 cells/µl** | **CD4^+^ >200 cells/µl** | **Association of inflammatory markers with NCI/HIVE** | |
| --- | --- | --- | --- | --- |
|  |  |  | **Yes** | **No** |
| [81] | X |  | X |  |
| [29] | X |  | X |  |
| [83] | X |  | X |  |
| [85] | X |  |  | X |
| [90] | X |  | X |  |
| [93] | X |  | X |  |
| [94] | X |  | X |  |
| [13] | X |  | X |  |
| [100] | X |  | X |  |
| [102] | X |  | X |  |
| [103] | X |  |  | X |
| [106] | X |  | X |  |
| [107] | X |  | X |  |
| [108] | X |  | X |  |
| [109] | X |  | X |  |
| [110] | X |  | X |  |
| [30] |  | X | X |  |
| [113] | X |  | X |  |
| [119] | X |  | X |  |
| [120] | X |  | X |  |
| [121] | X |  |  | X |
| [14] | X |  | X |  |
| [124] | X |  | X |  |
| [126] | X |  | X |  |
| [127] | X |  | X |  |
| [131] | X |  | X |  |

**Abbreviations:** HIVE: HIV encephalitis; NCI: Neurocognitive impairment
