# Supplementary material for: The relationship between HIV‐1 neuroinflammation, neurocognitive impairment and encephalitis pathology: A systematic review of studies investigating post‐mortem brain tissue
Source: Rev Med Virol. 2024 Jan 27;34(1):e2519. doi: 10.1002/rmv.2519 (PMC10909494; doi:10.1002/rmv.2519)
Supplement: Supplementary file 5 — Table S4 [file RMV-34-e2519-s004.docx]

**Supplementary Table 4**: Neuroinflammatory gene transcript levels associated with neurocognitive impairment and/or HIV encephalitis in PLWH

| Marker | Association with NCI/HIVE | | Marker levels | Brain section | NCI/HIVE | Reference |
| --- | --- | --- | --- | --- | --- | --- |
|  | Yes | No |  |  |  |  |
| Bone marrow stromal cell antigen 2 | ✔ |  | ↑ | FC | HIVE | [105] |
| CCL2 | ✔ |  | ↑ | Cerebral WM | NCI | [86] |
|  |  | ✔ |  | Right dorsolateral and MFC. | NCI | [103] |
|  |  |  |  | Caudate and ACC | NCI | [131] |
| CCL3 |  | ✔ |  | Right dorsolateral and MFC. | NCI | [103] |
| CCR5 |  | ✔ |  | Caudate and ACC | NCI | [131] |
| CX3CL1 | ✔ |  | ↑ | FC | NCI | [108]( |
| CXCL12 |  | ✔ |  | Right dorsolateral and MFC. | NCI | [103] |
|  |  | ✔ |  | FC | HIVE | [116] |
| CXCR1 |  | ✔ |  | Right dorsolateral and MFC. | NCI | [103] |
| CXCR4 |  | ✔ |  | Caudate and ACC | NCI | [131] |
| CD14 | ✔ |  | ↑ | Cortex, BG, WM and  cerebellum | HIVE | [135] |
| Gal-9 | ✔ |  | ↑ | FWM | HIVE | [113] |
| h2-microglobulin | ✔ |  | ↑ | FC | HIVE | [105] |
| HLA-DR3 |  | ✔ |  | N/A | HIVE | [118] |
| IgG heavy constant-g3 | ✔ |  | ↑ | FC | HIVE | [105] |
| IL-1 | ✔ |  | ↓ | FC | HIVE | [90] |
| IL-1β | ✔ |  | ↑ | FC | NCI and HIVE | [99] |
|  |  |  |  | FC | NCI | [83] |
|  |  |  |  | FC and BG cerebellum, and WM | NCI | [123] |
|  |  |  |  | FC | NCI | [14] |
|  |  |  |  | Frontal Lobe | NCI | [106] |
|  |  | ✔ |  | Cortex, BG, WM and  cerebellum | HIVE | [135] |
| IL-2 |  | ✔ |  | Cortex, subcortical, and deep WM from the right frontal lobe and globus pallidus from the BG | NCI | [127] |
| IL-4 |  | ✔ |  | Cortex, subcortical, and deep WM from the right frontal lobe and globus pallidus from the BG | NCI | [127] |
| IL-6 | ✔ |  | ↓ | FC | HIVE | [90] |
|  |  | ✔  ✔ |  | Right dorsolateral and MFC. | NCI | [103] |
|  |  |  |  | Cortex, subcortical, and deep WM from the right frontal lobe and globus pallidus from the BG | NCI | [127] |
| IL-8 | ✔ |  | ↑ | FC and BG | HIVE | [130] |
| IL-10 | ✔ |  | ↑ | FC | NCI | [83] |
|  |  | ✔ |  | Cortex, subcortical, and deep WM from the right frontal lobe and globus pallidus from the BG | NCI | [127] |
| IL-33 |  | ✔ |  | FC | NCI | [99] |
| Major histocompatibility complex (MHC) classes 1A, C, F, | ✔ |  | ↑ | FC | HIVE | [105] |
| MIP- 1α | ✔ |  | ↑ | Brain and spinal cord tissue | NCI | [107] |
| MIP- 1β | ✔ |  | ↑ | Brain and spinal cord tissue | NCI | [107] |
| MIG-2 |  | ✔ |  | Cortex, subcortical, and deep WM from the right frontal lobe and globus pallidus from the BG | NCI | [127] |
| TGF-β1 | ✔ |  | ↑ | FC | NCI | [92] |
|  |  | ✔ |  | Cortex, subcortical, and deep WM from the right frontal lobe and globus pallidus from the BG | NCI | [127] |
| TGF-β2 | ✔ |  | ↑ | FC | NCI | [92] |
|  |  | ✔ |  | Cortex, subcortical, and deep WM from the right frontal lobe and globus pallidus from the BG | NCI | [127] |
| TNF2 |  | ✔ |  | N/A | HIVE | [118] |
| TNF-α | ✔ |  | ↑ | FC | NCI | [13] |
|  |  |  |  | Cerebrum | NCI | [107] |
|  |  |  |  | Cortex, BG, WM and  cerebellum | HIVE | [135] |
|  |  |  |  | Cortex, subcortical, and deep WM from the right frontal lobe and globus pallidus from the BG | NCI | [127] |
|  |  |  |  | Frontal Lobe | NCI | [106] |
|  |  | ✔ |  | Right dorsolateral and MFC. | NCI | [103] |
|  |  |  |  | FC | HIVE | [90] |
| TNFRI |  | ✔ |  | Frontal lobe and subcortical WM | HIVE | [122] |
| TNFRII |  | ✔ |  | Frontal lobe and subcortical WM | HIVE | [122] |

**Abbreviations:** ACC: anterior cingulate cortex, AM: amygdala, BG: Basal ganglia, CB: cerebellum, CCL: C-C chemokine ligand, CCR: chemokine receptor, CD: Cluster of differentiation CXCL: Chemokine (C-X-C motif) ligand, CXCR: C-X-C chemokine receptor type, FC: frontal cortex, FWM: frontal white matter, Gal: Galectin, HIVE: HIVE encephalitis, HLA-DR: Human Leukocyte Antigen – DR isotype (HLA-DR), IL: Interleukin, MED: medulla, MFC: Midfrontal cortex, MHC-II: Major histocompatibility class II, MIP: macrophage inflammatory protein, NCI: neurocognitive(ly) impaired, PLWH: people living with HIV, TGF: Transforming growth factor, TNF: Tumor necrosis factor, TNFR: Tumor necrosis factor receptor and WM: White matter
