# Supplementary material for: The relationship between HIV‐1 neuroinflammation, neurocognitive impairment and encephalitis pathology: A systematic review of studies investigating post‐mortem brain tissue
Source: Rev Med Virol. 2024 Jan 27;34(1):e2519. doi: 10.1002/rmv.2519 (PMC10909494; doi:10.1002/rmv.2519)
Supplement: Supplementary file 6 — Table S5 [file RMV-34-e2519-s006.docx]

**Supplementary Table 5:** Inflammatory protein cytokine levels associated with neurocognitive impairment or HIV encephalitis in PLWH.

| **Marker** | **Association with NCI/HIVE** | | **Marker levels** | **Brain section** | **NCI/HIVE** | **Reference** |
| --- | --- | --- | --- | --- | --- | --- |
|  | **Yes** | **No** |  |  |  |  |
| CCL2 | ✔ |  | ↑ | N/A | HIVE | [110] |
| CD8 | ✔ |  | ↑ | BG and parietal regions | NCI | [133] |
| CD14 | ✔ |  | ↑ | Cerebral WM and adjacent cortex and/or BG | HIVE | [87] |
|  |  |  |  | N/A | HIVE | [95] |
|  |  | ✔ |  | N/A | HIVE | [96] |
| CD16 | ✔ |  | ↑ | N/A | HIVE | [95] |
|  |  |  |  | N/A | HIVE | [96] |
|  |  | ✔ |  | FWM and BG | HIVE | [124] |
| CD40 | ✔ |  | ↑ | FC | HIVE | [114] |
| CD45 | ✔ |  | ↑ | Cerebral WM and adjacent cortex and/or BG | HIVE | [87] |
|  |  | ✔ |  | FC | HIVE | [105] |
|  |  |  |  | FC | NCI | [30] |
| CD45RA |  | ✔ |  | Frontal lobe | HIVE | [88] |
| CD45RB | ✔ |  | ↑ | Frontal lobe | HIVE | [88] |
| CD45RC |  | ✔ |  | Frontal lobe |  | [88] |
| CD45R0 | ✔ |  | ↑ | Frontal lobe | HIVE | [88] |
|  |  |  |  | Hippocampus | HIVE | [112] |
| CD68 | ✔ |  | ↑ | GM | HIVE | [82] |
|  |  |  |  | Hippocampus | HIVE | [29] |
|  |  |  |  | Cortical WM, the deep GM, and, to a lesser extent, the cortical GM | HIVE | [89] |
|  |  |  |  | N/A | HIVE | [96] |
|  |  |  |  | Hippocampus | HIVE | [112] |
|  |  |  |  | BG, parietal and MB | NCI | [133] |
|  |  | ✔ |  | BG | HIVE | [29] |
|  |  |  |  | Frontal lobe | HIVE | [82] |
|  |  |  |  | FC | NCI | [30] |
|  |  |  |  | FWM and BG | HIVE | [124] |
| CX3CL1 | ✔ |  | ↑ | FC | NCI | [108] |
| CXCL12 |  | ✔ |  | MFC | HIVE | [101] |
|  |  |  |  | BG and FC | HIVE | [116] |
| Galectin-9 |  | ✔ |  | FC and white matter | HIVE | [113] |
| GFAP | ✔ |  | ↑ | Frontal lobe | HIVE and NCI | [81] |
|  |  |  |  | FC | HIVE | [94] |
|  |  |  |  | MFG, parietal cortex and CB | NCI | [71] |
|  |  |  |  | N/A | HIVE | [110] |
|  |  |  |  | Hippocampus | HIVE | [111] |
|  |  |  |  | Entorhinal cortex, hippocampus and subcortical WM t | NCI | [126] |
|  |  |  |  | FC | HIVE | [128] |
|  |  | ✔ |  | BG and hippocampus | HIVE | [29] |
|  |  |  |  | Hippocampus, putamen, and internal capsule, FC | NCI | [85] |
|  |  |  |  | Cortex | HIVE | [97] |
|  |  |  |  | Frontal lobe and BG | NCI | [100] |
|  |  |  |  | OC | NCI | [102] |
|  |  |  |  | FC | HIVE | [105] |
|  |  |  |  | FC | NCI | [30] |
|  |  |  |  | Frontal lobe | NCI | [119] |
|  |  |  |  | Cortical WM (from the BG including the putamen and  pallidum, and from the deep WM of the centrum ovale) | NCI | [120] |
|  |  |  |  | Frontal lobe | HIVE | [125] |
|  |  |  |  | FC and BG | NCI | [126] |
| HAM-56 | ✔ |  | ↑ | MFG and the BG | NCI | [98] |
| HLA-DR |  | ✔  ✔ |  | Temporal cortex (TC), BG, and brain stem | HIVE | [79] |
|  |  |  |  | Hippocampus, putamen, and internal capsule, FC | NCI | [85] |
|  |  |  |  | Cortical WM, the deep GM, and, to a lesser extent, the cortical GM | HIVE | [89] |
|  |  |  |  | Cortex | HIVE | [97] |
|  |  |  |  | Parietal cortex | NCI | [71] |
|  |  |  |  | OC | NCI | [102] |
|  |  |  |  | Hippocampus | HIVE | [111] |
|  |  |  |  | FC | NCI | [30] |
|  |  |  |  | BG and Hippocampus | HIVE | [29] |
|  | ✔ |  | ↑ | N/A | HIVE | [95] |
|  |  |  |  | MFG and CB | NCI | [71] |
|  |  |  |  | N/A | HIVE | [110] |
| Iba-1 |  | ✔ |  | Hippocampus, putamen, and internal capsule, FC | NCI | [85] |
|  | ✔ |  | ↑ | Frontal lobe WM, FC and BG | HIVE and NCI | [91] |
|  |  |  |  | FC | HIVE | [94] |
| IL-1 | ✔ |  | ↑ | FC or BG (putamen) | HIVE | [134] |
|  |  | ✔ |  | Frontal cortex (FC), caudate nucleus, insular cortex, Basal Ganglia (BG), thalamus, hypothalamus, hippocampus, superior cerebellum (CB), midbrain (MB), pons (PN) and medulla (MED) | HIVE | [78] |
|  |  |  |  | Temporal cortex (TC), BG, and brain stem | HIVE | [79] |
| IL-1α | ✔ |  | ↑ | Cortex and White matter (WM) | HIVE | [80] |
| IL-1β | ✔ |  | ↑ | FC | HIVE | [99] |
|  |  |  |  | Frontal lobe and pons | HIVE | [129] |
|  |  |  |  | FC | HIVE | [104] |
| IL-2 |  | ✔ |  | MFC, cortical and subcortical regions | HIVE | [104] |
| IL-3 |  | ✔ |  | Frontal cortex (FC), caudate nucleus, insular cortex, Basal Ganglia (BG), thalamus, hypothalamus, hippocampus, superior cerebellum (CB), midbrain (MB), pons (PN) and medulla (MED) | HIVE | [78] |
| IL-4 | ✔ |  | ↓ | Cortex and White matter (WM) | HIVE | [80] |
| IL-6 | ✔ |  | ↑ | FC | HIVE | [90] |
|  |  | ✔ |  | Frontal cortex (FC), caudate nucleus, insular cortex, Basal Ganglia (BG), thalamus, hypothalamus, hippocampus, superior cerebellum (CB), midbrain (MB), pons (PN) and medulla (MED) | HIVE | [78] |
|  |  |  |  | Cortex and White matter (WM) | HIVE | [80] |
| IL-16 | ✔ |  | ↑ | Cerebral WM and adjacent cortex or BG | HIVE | [132] |
| IL-33 |  | ✔ |  | FC | HIVE | [99] |
| MAP2 | ✔ |  | ↑ | OC | NCI | [102] |
|  |  |  |  | Right dorsolateral and MFC. | NCI | [103] |
| MIP- 1α | ✔ |  | ↑ | N/A | HIVE | [110] |
| MIP-1β | ✔ |  | ↑ | N/A | HIVE | [110] |
| MMP-2 | ✔ |  | ↑ | FC and BG cerebellum, and WM | NCI | [123] |
| OPN | ✔ |  | ↑ | Occipital lobes | NCI | [84] |
|  |  | ✔ |  | Occipital lobes | NCI | [121] |
| CCL5 | ✔ |  | ↑ | Frontal lobe and BG | HIVE | [136] |
|  |  |  |  | N/A | HIVE | [110] |
| S-100A8 | ✔ |  | ↑ | deeper midline and mesial temporal structure | NCI | [133] |
| TGF-β1 | ✔ |  | ↑ | FC | HIVE | [104] |
| TGF-β2 | ✔ |  | ↑ | FC | NCI | [92] |
| TIMP-1 | ✔ |  | ↓ | FC and BG cerebellum, and WM | NCI | [123]) |
| TNF-α | ✔ |  | ↓ | Cortex and White matter (WM) | HIVE | [80] |
|  | ✔ |  | ↑ | frontal deep WM and the BG | NCI | [115] |
|  |  |  |  | the deep WM and the MFC | HIVE | [120] |
|  |  |  |  | Frontal lobe and pons | HIVE | [137] |
|  |  |  |  | BG | HIVE | [78] |
|  |  |  |  | Frontal cerebral cortex and BG | NCI | [100] |
|  |  | ✔ |  | Temporal cortex (TC), BG, and brain stem | HIVE | [79] |
|  |  |  |  | Frontal lobe and subcortical WM | NCI | [122] |
| TNFRI |  | ✔ |  | Frontal lobe and subcortical WM | NCI | [122] |
| TNFRII |  | ✔ |  | Frontal lobe and subcortical WM | NCI | [122] |
| TRAIL | ✔ |  | ↑ | FC and BG | HIVE | [117] |

**Abbreviations:** BG: Basal ganglia, CB: cerebellum, CCL: C-C chemokine ligand, CD: Cluster of differentiation CXCL: Chemokine (C-X-C motif) ligand, FC: frontal cortex, FWM: frontal white matter, Gal: Galectin, GFAP: Glial fibrillary acidic protein, HIVE: HIVE encephalitis, HLA-DR: Human Leukocyte Antigen – DR isotype (HLA-DR), Iba-1: ionized calcium-binding adapter molecule, IL: Interleukin, MB: midbrain, MED: medulla, MFC: Midfrontal cortex, MIP: macrophage inflammatory protein, MMP: Matrix metalloproteinases, NCI: neurocognitive(ly) impaired, OC: occipital cortex, OPN: Osteopontin, PLWH: people living with HIV, PN: pons, TC: temporal cortex, TGF: Transforming growth factor, TIMP: tissue inhibitors of metalloproteinases, TNF: Tumor necrosis factor, TNFR: Tumor necrosis factor receptor and WM: White matter
